# Carbamoylation as an Effective Tool in the Analysis of the Soman Nerve Agent Marker Pinacolyl Alcohol in Soil Matrices by EI-GC-MS and LC-HRMS

**DOI:** 10.1021/acsomega.6c00271

**Published:** 2026-05-01

**Authors:** David Baliu-Rodriguez, David S. Cho, Adele F. Panasci-Nott, Saphon Hok, Alexander K. Vu, Mark L. Dreyer, Carlos A. Valdez

**Affiliations:** †Physical and Life Sciences Directorate, ‡Biosciences and Biotechnology Division, §Forensic Science Center, and ∥Global Security Directorate, 4578 Lawrence Livermore National Laboratory, Livermore, California 94550, United States

## Abstract

Pinacolyl alcohol (PA) is a Schedule 2 chemical commonly featured in most proficiency tests (PTs) administered by the Organisation for the Prohibition of Chemical Weapons (OPCW) due to its direct as well as forensic link to the nerve agent Soman. Therefore, its detection by Chemical Weapons Convention (CWC) inspection teams during on-site investigations is a strong indicator of the past or latent presence of Soman in the environment. Its small molecular weight (102), early elution time, and poor ionization profile make PA a challenging analyte to detect particularly at low concentrations (∼1–10 μg/g). In this work, 1,1′-carbonyldimidazole (CDI) has been used to effectively modify PA for the first time in two different soil matrices (Virginia type A soil and silt sediment) at two separate concentrations (1 and 10 μg/g) for its subsequent detection by EI-GC-MS and LC-HRMS methods. For the EI-GC-MS analysis, the PA carbamate derivative (PIC) exhibits improved chromatography relative to PA such as improved peak shape, increased molecular weight (196 for PIC and 102 for PA) and increased retention time (16.9 min for PIC and ∼4.1 min for PA). In addition, the derivatization also improves the detection of PA by LC-HRMS as the PIC product possesses protonation sites (i.e., imidazole ring) relative to none exhibited by PA. More importantly, the carbamoylation proceeds under mild conditions (55 °C, no base) and rapidly (3 h), characteristics that make it an appealing protocol for the analysis of PA during OPCW PTs or real case scenarios particularly in instances where it is present at low concentrations. It is anticipated that the protocol can be applied to the forensic analysis of this important Soman marker in various environmental matrices.

## Graphical abstract

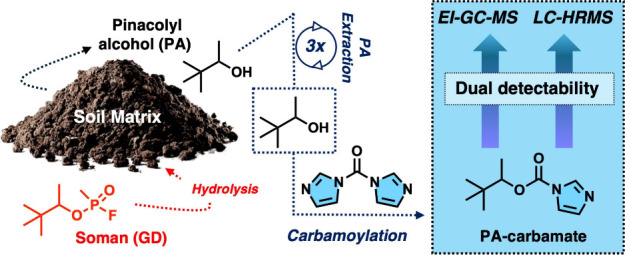

## 1 Introduction

Organophosphorus-based nerve agents (OPNAs) are one of the most feared members in the growing list of chemical warfare agents (CWAs) due to their extremely high toxicity.^1,2^ Their deliberate use against military targets during the Iraq-Iran conflict^3,4^ and against civilian populations, as highlighted by the Tokyo subway attack by the Aum Shinrikyo^5,6^ along with the chemical attacks in Ghouta and Khan Shaykhun in Syria,^7,8^ have caused constant global concern in the past 40 years. As a result of this, several research efforts have experienced increased activity that include the development of protective materials,^9,10^ newer broad-spectrum antidotes,^11–17^ and more efficient ways for their degradation.^18–21^ In parallel fashion, significant effort continues to be invested in the direct detection and analysis of OPNAs by various analytical means, as well as their degradation products in the environment for chemical forensics applications.^22–24^ It is in the field of chemical forensics that the efficient detection of these toxic chemicals or their degradation products becomes a key capability for Chemical Weapons Convention (CWC) inspection teams deployed to investigate and validate reports involving the alleged use of OPNAs in a particular location.^25^

Often, OPNA degradation products are polar and very difficult to analyze by conventional GC-MS and LC-MS methods without prior derivatization of the sample, particularly in instances where these are present at low concentrations.^26–29^ One such product is pinacolyl alcohol (PA, 3,3-dimethyl-2-butanol) which is a building block in the synthesis, and naturally a degradation byproduct, of the nerve agent Soman (GD, 1-methyl-2,2-dimethylpropyl methylphosphonofluoridate) (Figure 1a). Its low molecular weight (*m*/*z* = 102), early elution time (during GC-MS analysis), and poor ionization in electrospray-based LC-MS methods make PA a difficult analyte to detect by routine GC-MS and LC-MS methods. For these reasons, detection of PA is often challenging in proficiency tests (PTs) administered by the Organisation for the Prohibition of Chemical Weapons (OPCW), particularly in matrices where more abundant, coeluting interferences are deliberately introduced.

**Figure 1:**
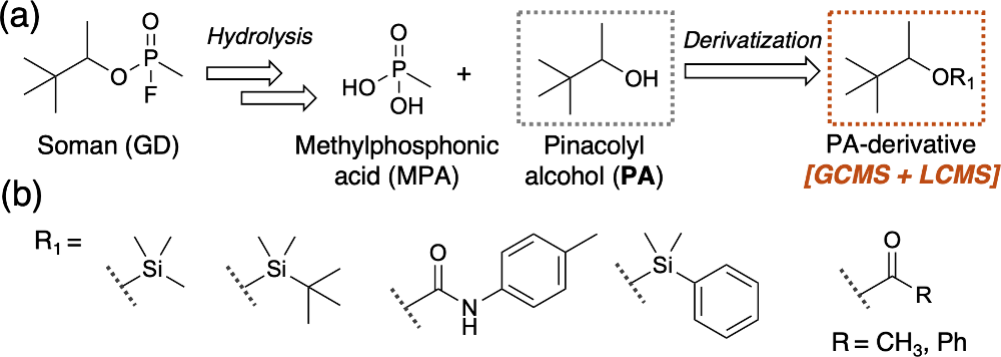
(a) Pinacolyl alcohol (PA), degradation product from the nerve agent Soman (GD) along with methylphosphonic acid (MPA). PA can be derivatized to yield analogs that can be detected by GC-MS and LC-MS. (b) Derivatization strategies for PA including silylation using silyl chlorides as well as reagents like BSTFA and MTBSTFA to yield trimethylsilylated, *tert*-butyldimethylsilylated, and phenyldimethylsilylated derivatives; carbamate formation using *p*-tolylisocyanate; and acylation using acetic anhydride and benzoyl chloride.

To this end, several methods for the derivatization of PA have been devised with the goal of improving its detection and analysis. These chemical modifications seek to improve its chromatographic profile by drastically increasing its molecular weight and consequently shifting its column retention time away from other coeluting matrix interferences (Figure 1b). One of the most employed derivatization methods for PA involves silylation^30,31^ in the form of the trimethylsilyl (TMS)^32^ and *tert*-butyl-dimethylsilyl (TBDMS)^33^ ethers. Although these are very efficient at providing derivatives that can be detected by GC-MS, as well as by LC-MS, their poor stability under mildly basic or acidic conditions prevents their widespread use during sample preparation techniques. Efforts on improving the stability of silyl derivatives led to the development of the phenyldimethylsilyl derivatization protocol using *N*-methylimidazole as a catalyst by Albo et al.,^34^ allowing the silylation to take place at ambient temperature and producing a PA derivative with increased retention time and stability relative to its TMS counterpart.^35^ Another PA derivatization method involves the use of *p*-tolylisocyanate (PTI) which generates a carbamate of PA featuring superior stability relative to silyl groups and an extended retention time in the GC column.^36,37^ An additional method for the derivatization of PA, involved its acetylation and benzoylation to yield esters that proved to be extremely useful in modifying its retention time significantly for its detection when present at low concentrations in glycerol-rich matrices.^38,39^

We have focused our efforts on developing derivatization techniques that are applicable to both GC-MS and LC-MS, however, with more particular emphasis on the former as samples that can be detected by GC-MS often will be successfully detected by the more sensitive LC-MS technique. Furthermore, during OPCW PTs and real sample analysis, having two orthogonal detection techniques provides us with a higher level of confidence in chemical identification using one derivatized version of an analyte. However, as field analysis by CWC inspection teams using portable GC-MS units^40–42^ offers the ability to analyze a sample on site, it is important for the team analysts to possess efficient derivatization techniques to convert polar analytes into products that are amenable for GC detection.^43^ The reason for this is that CWAs are highly reactive and therefore do not exhibit long persistence in the environment, rapidly degrading to polar products that are notoriously difficult to detect by GC-MS. For example, as shown in Figure 1a, Soman (GD) will undergo complete degradation to ultimately yield methylphosphonic acid (MPA) and pinacolyl alcohol (PA), two polar compounds that are notoriously difficult to detect by GC-MS means. Another convenient facet of GC-MS analysis comes from the ability of matching unknown analytes’ mass spectra to the instrument’s internal mass spectral libraries (e.g., NIST, OCAD) that become an invaluable tool in the preliminary field identification of such analyte.^44–46^ Naturally, after the initial putative identification of an unknown analyte by the instrument’s internal library, the next step involves the acquisition or the synthesis of a standard for complete corroboration of the unknown’s identity.

In this work, 1,1′-carbonyldimidazole (CDI) is employed for the first time as an effective reagent for the modification and analysis of PA in two types of soil matrices. The modification results in the conversion of PA into a derivative (PIC) possessing structural features that make it a suitable species for enhanced detection by GC-MS and LC-MS such as increased molecular weight (from *m*/*z* = 102 to *m*/*z* = 196), increased retention time in GC-MS analysis from 4.1 min for PA (broad peak) to 16.9 min for PIC (sharp peak), and the presence of an imidazolyl carbamate moiety which in itself is a UV detection tag and a site for protonation (at the imidazolyl N3) during LC-HRMS analysis.^47^ An additional benefit for using 1,1′-carbonyldimidazole (CDI) is that the generated PIC carbamate exhibits superior stability, toward both hydrolysis (i.e., humidity) and thermal degradation during GC analysis relative to the commonly used PA derivative, namely, trimethylsilyl pinacolyl alcohol (PA-TMS). Comparative experiments evaluating the stability of PIC and PA-TMS have been conducted and are included in the Supporting Information (pages S3–S6). The two soil matrices used in this study are Virginia type A (VA) soil and silt sediment and were chosen for their different physical as well as chemical properties.^48^ Whereas VA soil possesses a high total organic content and moisture, silt sediment features silicon dioxide-based fine particles mixed with clay, both leading to the development of unique extraction and derivatization methods. The protocol used in this work is outlined in Figure 2, and it bears a certain degree of similarity to the one used by our laboratory for the analyses of soil matrices featured in OPCW PTs. Thus, the protocol involves the initial extraction of PA from each soil using an organic solvent. During this first step, each soil matrix was found to require different extraction solvents, with dichloromethane (DCM) found to be optimal for VA and 1-chlorobutane (1-CB) found to be the most efficient for extracting PA from silt sediment (Figure 2). However, chloroform (CHCl_3_) was found to be a unifying solvent for the extraction of both matrices, with extraction efficiencies for PA rivaling those exhibited by DCM and 1-CB. After PA extraction (each soil undergoes a round of three extractions), the extracts are evaporated and the residue derivatized using CDI. The final, derivatized samples are then analyzed by electron impact gas chromatography–mass spectrometry (EI-GC-MS) as well as liquid chromatography–high resolution mass spectrometry (LC-HRMS) for PT reporting purposes (Figure 2).

**Figure 2:**
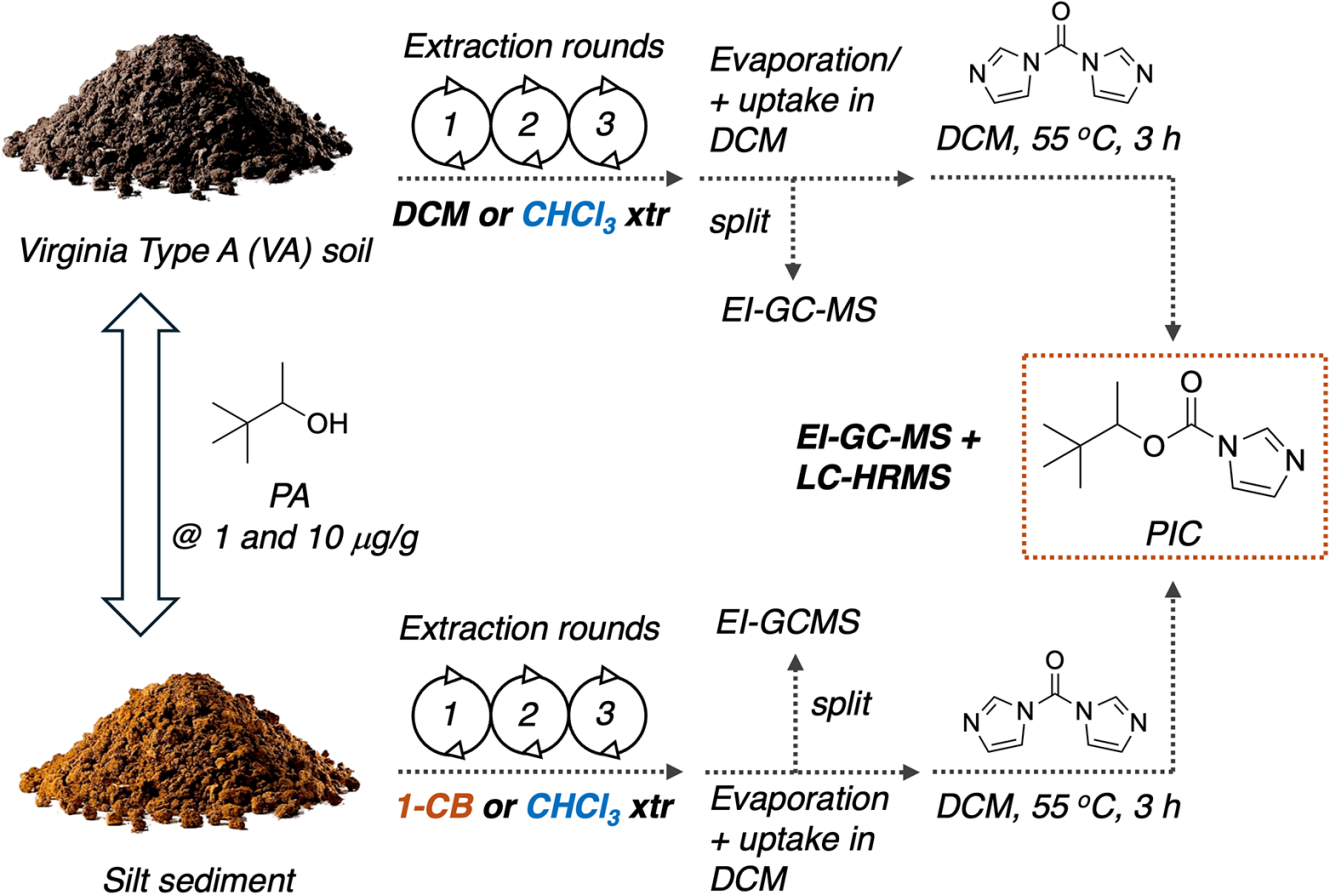
Outline of the protocol described in this work involving the analysis of PA in two soils: Virginia Type A (VA) soil and silt sediment when spiked at two concentrations (1 and 10 μg/g). PA was extracted using dichloromethane or chloroform from the VA soil while 1-chlorobutane (1-CB) or chloroform was used to extract PA from silt. Each extraction was composed of three rounds, and after collection of all extracts and evaporation, the residues are taken up in DCM and split into one for direct analysis by EI-GC-MS and another one for derivatization with CDI followed by analysis by EI-GC-MS and LC-HRMS. (Soil photograph courtesy of iStock photo (credit: malerapaso). Copyright 2025.)

## 2 Results and Discussion

### 2.1 Selection of PA as a Marker of Interest

PA was chosen for its direct link to the OP-based nerve agent Soman. As Soman undergoes hydrolysis, environmentally or biologically, it yields PA among other important markers such as pinacolyl methylphosphonic acid and methylphosphonic acid (MPA) (Figure 1a). Therefore, PA can serve as an indicator for the latent or previous presence of Soman in a matrix. PA is classified by the Organisation for the Prohibition of Chemical Weapons (OPCW) as a Schedule 2 substance defining it as a “precursor in one of the chemical reactions at the final stage of formation of a chemical listed in Schedule 1 (i.e., nerve agent).” For these reasons, PA has become a commonly featured analyte in PTs administered by OPCW, often spiked in low concentrations among more concentrated, deliberately added coeluting interferences. To this end, several methods for its analysis have been developed over the years to improve its detection by GC-MS as well as LC-MS means. Derivatization reactions, introduced in Figure 1b, have focused on improving PA’s GC-MS profile, unfortunately, not all of these amenable for subsequent LC-MS analysis, thus still requiring the analyst to conduct analysis on several samples. For this reason, one of the main goals of the protocol introduced herein is to produce a PA derivative in one sample that can be analyzed by GC-MS as well as LC-HRMS without wasting precious analysis time specifically during OPCW PTs that participating laboratories need to complete in 2 weeks.

### 2.2 Selection of Soil Matrices, Extraction Solvents, and Spiking Concentrations

Part of the CWC inspection team’s duties includes the collection of environmental samples in the vicinity or at the site of alleged OPNA use. Collected samples often involve soil and liquid matrices found in the environment but can also include other matrices such as clothing or even vegetation. For our studies and as a means of using a representative soil matrix, Virginia Type A (VA) soil was chosen due to its standard use for extraction experiments among analytical laboratories since it contains organic components (total organic content, 2.6%) as well as a low clay concentration (7.5%). Another soil matrix chosen for the studies was silt sediment due to its different physical and chemical constitution from VA. Silt possesses the texture of a free-flowing soil and contains a higher clay concentration (25%), making the extraction and subsequent derivatization of analytes a challenging task. Therefore, both soil matrices are very different from each other not only chemically but also physically as VA is composed of particles in various aggregate sizes while silt is composed of uniform particles throughout.

Five solvents were screened for the extraction of PA from VA soil, and these included the following: dichloromethane (DCM), ethyl acetate (EtOAc), acetonitrile (ACN), 1-chlorobutane (1-CB), and chloroform (CHCl_3_). Dichloromethane was included in our panel, as it is a commonly employed solvent in GC-MS analyses due to its polarity and low boiling point (39.6 °C). Ethyl acetate, like DCM, is an alternate solvent that is used for GC-MS analyses, added in our panel for its moderate polarity (0.28 relative to 1 (water)), and possesses a low density (*d* = 0.90 g/mL) and an elevated boiling point (77 °C). Acetonitrile was used in our panel as it is a common solvent for analyses done by LC-MS methods as it possesses a high polarity (0.46 relative to 1 (water)) and aids in the solvation of polar compounds. Another solvent included was 1-chlorobutane due to its ability to extract organic compounds in a fashion similar to that of DCM but featuring a lower density (*d* = 0.88 g/mL), thus providing an alternate approach when performing the extraction section of the protocol. The last solvent included in the panel was chloroform (CHCl_3_), that like DCM is denser than water but possesses a higher boiling point (bp = 61.2 °C) in addition to increased lipophilicity which may aid in the extraction of PA from the matrices. Lastly, to mimic the scenario featured in OPCW PTs, PA was spiked in each soil matrix at two concentrations, 1 and 10 μg/g.

### 2.3 Extraction and Derivatization of PA in Virginia Type A Soil

Five solvents were screened for the extraction of PA from VA soil, and these included the following: dichloromethane (DCM), ethyl acetate (EtOAc), acetonitrile (ACN), 1-chlorobutane (1-CB), and chloroform (CHCl_3_). Figure 3a shows that DCM and CHCl_3_ are superior solvents for the extraction of PA from the VA soil matrix over EtOAc, ACN, and 1-CB for both spiked concentrations used in this work. It can be observed from the graphs in Figure 3a that the percentage of recovered PA from each extraction using DCM lies between 85 and 95% (blue bar) that of the standard solutions (purple bar), while the percentage for CHCl_3_ lies around the same vicinity (85–90%, blue bar), making it another great option for a PA-extracting solvent. Although it cannot be observed as a sharp peak, PA is extracted using DCM or CHCl_3_ efficiently as shown in Figure 3b, where its early elution time and broad nature of its peak can be perceived. After the extraction of the PA along with other organics from the soil matrix (3 × 1 mL), the DCM are combined, concentrated to ∼1 mL of total volume, treated with CDI, and heated to 55 °C for 3 h. The resulting mixture was then analyzed by EI-GC-MS. It was observed that, for both concentrations used, PA was successfully converted to PIC (Figure 3c–f). Although a direct analysis of the PIC can be performed at this stage by EI-GC-MS means, the actual mixture can be concentrated even further to effectively inject a more detectable quantity of the derivatized material. In the higher concentration case (i.e., 10 μg/g), PIC can be readily detected in the gas chromatogram using its characteristic retention time (RT = 16.9 min.) (Figure 3c and the inset). Standard analysis of the peak using background subtraction yielded a clean mass spectrum of the PIC (Figure 3d). In contrast, in the lower PA concentration sample (i.e., 1 μg/g), PIC was more difficult to locate due to its lower magnitude as well as the higher concentration of matrix components that also coeluted with the product (Figure 3e). However, application of the single ion extraction (SIE) analysis mode using *m*/*z* = 85 allows the detection of PIC as shown in the inset of Figure 3e and the collected mass spectrum (Figure 3f). The chosen total time for the derivatization reaction (e.g., 3 h) originated from a set of experiments designed to determine the time it took for the PA to be converted to PIC at 55 °C. It was found that within 2 h, virtually all of the PA had been converted to the PIC derivative when the reaction was carried out in CHCl_3_ extracts from each soil (Supporting Information, page S2).

**Figure 3:**
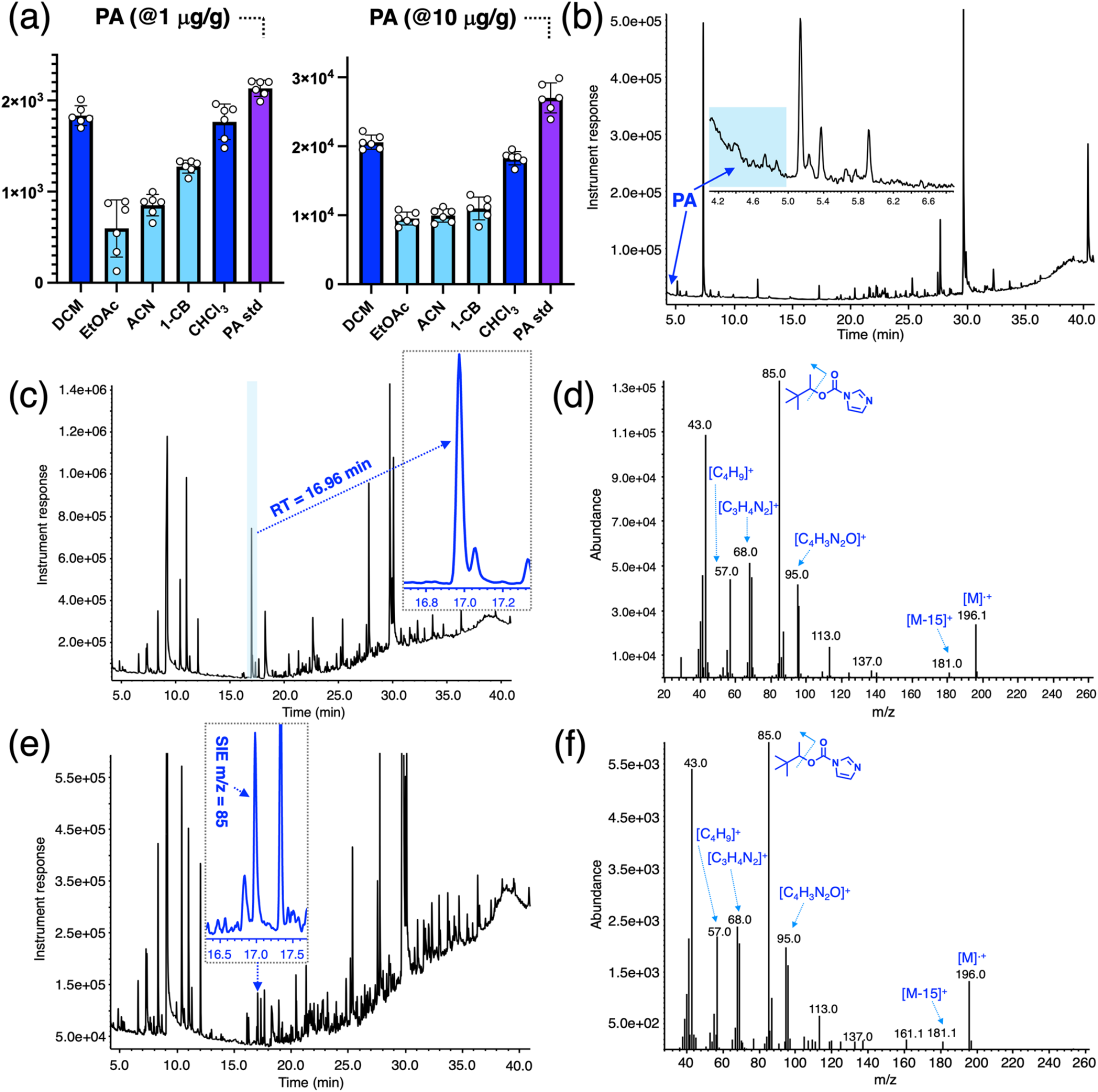
(a) Extraction efficiencies for PA from VA soil when spiked at 1 and 10 μg/g separately using five solvents: dichloromethane (DCM), ethyl acetate (EtOAc), acetonitrile (ACN), 1-chlorobutane (1-CB), and chloroform (CHCl_3_). Separate 1 and 10 μg/g standards were added to evaluate the efficiencies of each solvent at extracting the PA from the soil (average (*n* = 6) peak areas (±the standard deviation)). (b) Extraction of PA from VA soil using DCM when spiked at 10 μg/g. PA elutes broadly between 4 and 5 min (indicated by a blue arrow). (c) Reaction of the VA extract (PA at 10 μg/g) with CDI yielding the derivatized PIC product that can be observed with a retention time of 16.96 min (highlighted in light blue and expanded in inset). (d) Mass spectrum of PIC showing distinctive peaks and assigned structures and formulas of the fragments involved. (e) Reaction of the VA extract (with PA spiked at 1 μg/g) with CDI yielding PIC that can be detected by performing single ion extraction (SIE) analysis mode using *m*/*z* = 85 yielding several peaks in addition to the PIC product (inset). (f) Again, mass spectrum of PIC still revealing key fragments that can be used to identify the product.

### 2.4 Extraction and Derivatization of PA in Silt Sediment

Upon examination of the same panel of solvents used for the extraction of the VA soil, again it was found that 1-CB and CHCl_3_ are more efficient solvents for the extraction of PA over the other solvents assayed for silt sediment (Figure 4a). This was observed for the extraction of the PA at both concentrations, leading to extraction efficiencies almost mirroring the spiked PA standards included for comparison in Figure 4a. 1-CB was included in our studies because of its use as an efficient extraction solvent in biological applications, such as the extraction of organic molecules from blood, plasma, and urine samples.^49^ However, at this point, there is no clear evidence on why 1-CB would be a better extracting solvent than CHCl_3_ or DCM for PA when silt sediment is involved. One hypothesis is that 1-CB is more efficient at penetrating the clay-like matter making up the silt sediment than DCM, thus increasing the ability of PA to go into solution. Currently, we are assessing the ability of 1-CB to extract PA from other soil matrices for further comparison. However, in observing that CHCl_3_ possesses an outstanding extraction profile for PA and can be equally employed for its extraction from VA soil, from a convenience standpoint, it was selected as a unifying solvent for the extraction of PA from these soil matrices. After the extraction of the PA from the silt matrix (Figure 4b), CHCl_3_ was evaporated using a stream of nitrogen or a rotary evaporator (bp = 61.2 °C), and the residue was taken up in DCM and treated with CDI as a solution in DCM at 55 °C for 3 h. The resulting mixture was then analyzed by EI-GC-MS (Figure 4c–f). It was observed that for both sets of concentrations, PA was successfully converted to PIC. In the higher concentration case (i.e., 10 μg/g), PIC can be readily detected via its retention time (RT = 16.9 min, Figure 4c and inset). Standard analysis of the peak using background subtraction yielded a clean spectrum for the PIC (Figure 4d). With regard to the lower concentration sample (i.e., 1 μg/g) and in similar fashion to the VA soil case, PIC was more difficult to locate as a peak due to its low instrument response and coelution of interferences. However, by using the single ion extraction (SIE) analysis mode using *m*/*z* = 85, PIC can be unequivocally detected and identified, as shown in the inset of Figure 4e. The mass spectra collected from the analysis of the 1 μg/g sample (Figure 4f) were found to match the one for PIC.

**Figure 4:**
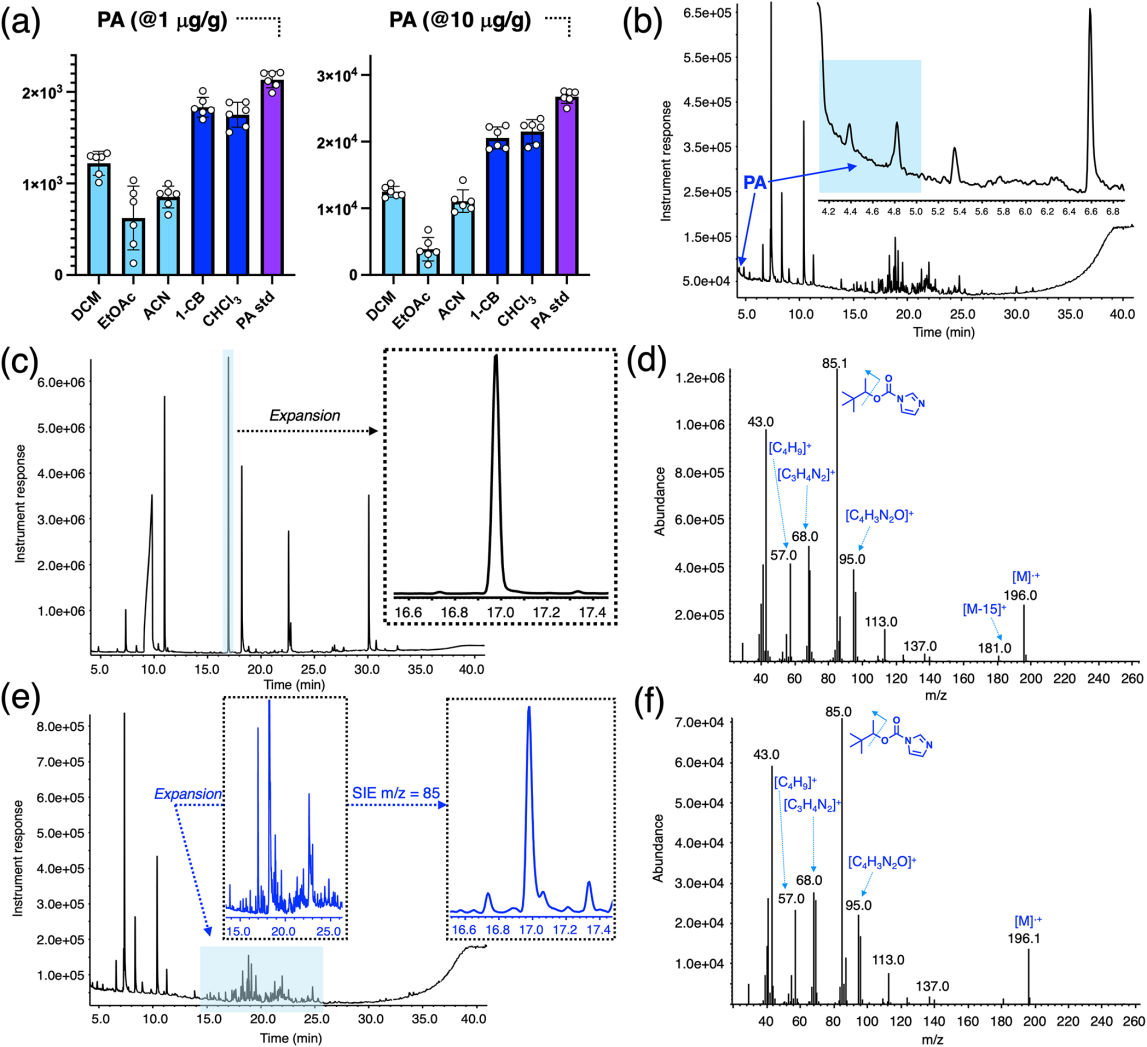
(a) Extraction efficiencies for PA from silt sediment when spiked at 1 and 10 μg/g separately using five solvents: dichloromethane (DCM), ethyl acetate (EtOAc), acetonitrile (ACN), 1-chlorobutane (1-CB), and chloroform (CHCl_3_). Separate 1 and 10 μg/g standards were added to evaluate the efficiencies of each solvent at extracting the PA from silt (average (*n* = 6) peak areas (±the standard deviation)). (b) Extraction of PA from silt using 1-CB when spiked at 10 μg/g. PA elutes broadly between 4 and 5 min (indicated by a blue arrow). (c) Reaction of the silt extract with CDI yielding the derivatized PIC product that can be observed with a retention time of 16.96 min (highlighted in light blue and expanded in inset). (d) Mass spectrum of PIC showing distinctive peaks and assigned structures and formulas of the fragments involved. (e) Reaction of the silt extract (with PA spiked at 1 μg/g) with CDI yielding PIC that can be detected by performing a single ion extraction (SIE) analysis mode using *m*/*z* = 85 yielding several peaks in addition to the PIC product (light blue area expanded in insets). (f) Mass spectrum of PIC still revealing key fragments that can be used to identify the product.

### 2.5 Analysis of PIC in Soil Samples by LC-HRMS

A central requirement from laboratories participating in OPCW PTs is the inclusion of secondary and tertiary data sets, preferably from orthogonal analytical methods, for the unequivocal and full characterization of a scheduled substance. Therefore, it is common that samples generated from an extraction/derivatization step during the PT are not only submitted for GC-MS analysis but also for analysis by complementary, hyphenated techniques such as GC-MS-MS (CI), GC-IR, GC-FPD, and LC-HRMS.^50^ To this end, the previously derivatized soil samples for both PA concentrations were subjected to LC-HRMS analysis as a way to confirm the presence and identity of PIC. Figure 5 shows the LC-HRMS analysis of all four samples (PA spiked at 1 and 10 μg/g in both VA and silt matrices) (Figure 5a) and the identification of PIC by its high resolution mass and characteristic fragmentation under positive mode electrospray ionization conditions (Figure 5b). It can be observed that even after performing a mass extraction analysis using a *m*/*z* range where the exact mass for PIC of 197.1284 lies with a small standard deviation window (*m*/*z* range of 197.1275–197.1295), several peaks possessing mass values within this range can be observed in the extracted chromatogram. However, only one possesses the mass within the theoretical mass value (within 5 ppm) leading to the proposed molecular formula involving C, H, N, and O atoms.

**Figure 5:**
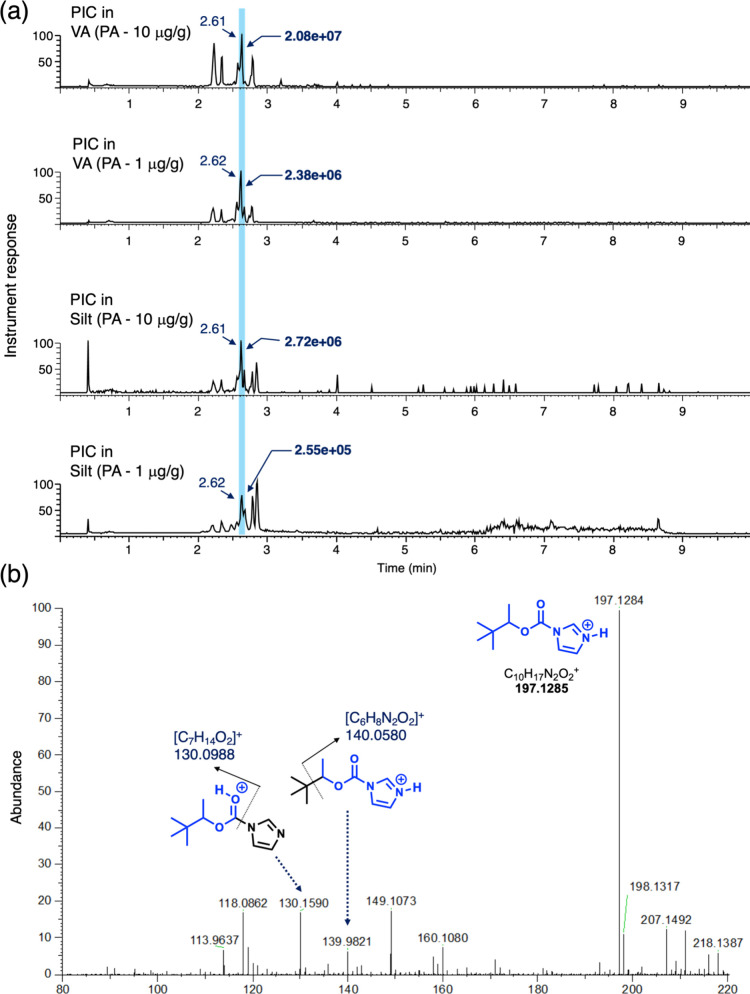
(a) LC-HRMS analysis of PIC in both soil matrices and at both concentrations. The PIC product elutes with a retention time of 2.62 min (indicated in each chromatogram by the light blue highlight). The chromatographs are the result of a single ion extraction using the exact mass *m*/*z* range (197.1275–197.1295). (b) Mass spectrum of PIC with partial assignments on some of the fragments showing the exact mass at *m*/*z* = 197.1284 (theoretical *m*/*z* = 197.1285).

In addition, MS-MS analysis was performed on the molecular ion (*m*/*z* = 197.1284) leading to the formation of three diagnostic fragments that aid in the structural confirmation of PIC (Figure 6). The first one of such fragments is *m*/*z* = 113.0343 (calculated *m*/*z* = 113.0346, mass error of 2.65 ppm) arising from a molecular formula of [C_4_H_5_N_2_O_2_]^+^ that can be envisioned to originate from fragmentation of PIC via a mechanism reminiscent of the McLafferty fragmentation (Figure 6). A second identifiable fragment is the one with a *m*/*z* = 85.1010 (calculated *m*/*z* = 85.1012, mass error of 2.35 ppm) arising from a molecular formula of [C_6_H_13_]^+^. This fragment can be envisioned to originate from heterolytic scission of the C–O bond connecting the imidazolyl carbamate to the alkyl group portion of the pinacolyl alcohol moiety (Figure 6). This fragmentation initially would lead to the secondary C_6_H_13_^+^ carbocation that would undergo methyl migration or shift to create a more stable tertiary carbocation with the same molecular formula (Figure 6). The last and third fragment is the one with a *m*/*z* = 69.0446 (calculated *m*/*z* = 69.0447, mass error of 1.45 ppm) arising from a molecular formula of [C_3_H_5_N_2_]^+^ that can be envisioned to originate from the [C_4_H_5_N_2_O_2_]^+^ fragment via a process that involves hydrogen transfer to the imidazole ring with concomitant CO_2_ loss (Figure 6). Collectively, the LC-HRMS and the MS-MS data obtained for PIC corroborate its structure and separate it from other potential products exhibiting similar exact masses. The ability to detect PIC by both analytical techniques (EI-GC-MS and LC-MS) is what makes this specific derivatization approach a useful tool for the analysis and detection of PA particularly during OPCW PTs. We anticipate the wide use of this derivatization approach in the analysis of this Soman marker in challenging matrices where other, established techniques such as silylation or acylation are not efficient at yielding observable derivatives.

**Figure 6:**
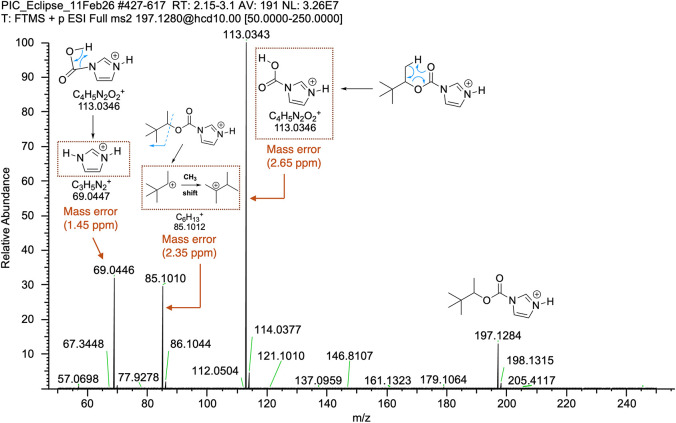
MS-MS spectrum of PIC using HCD fragmentation of the *m*/*z* = 197.1284 ion leading to three diagnostic fragments for the structure elucidation of the imidazolyl carbamate derivative. Possible mechanisms of formation for all three main fragments are denoted with blue arrows. The fragment responsible for the observed exact mass is boxed by the dotted lines, and their calculated, theoretical masses along with their mass errors are provided below their structures.

### 2.6 Determination of LOD and LOQ Values for PIC in Both Soils

Limit of detection (LOD) and limit of quantitation (LOQ) values for the PIC product were determined by preparing two stock solutions for PIC (100 μg/mL) in concentrated DCM extracts of each soil matrix (VA soil and silt sediment). Dilution of these solutions with additional, corresponding soil extracts was carried out until the S/N ratios fell between 5 and 20. The S/N ratios were calculated using the *m*/*z* = 85 ion (base peak for PIC). The calculated LOD and LOQ values for PIC in Virginia type A extract were found to be 4.9 and 16.5 ng/mL, respectively, while for silt sediment these were found to be 8.3 and 27.8 ng/mL, respectively (Supporting Information, pages S18–S19).

### 2.7 Comparative Studies on Carbamoylation and Silylation

Additional comparative studies were carried out on PA-spiked (20 μg/mL) sandy loam soil DCM extracts. The studies were aimed to evaluate the advantage of performing the carbamoylation derivatization of PA over other, well-established silylation methods like trimethylsilylation using BSTFA or phenyldimethylsilylation using phenyldimethylsilyl chloride and *N*-methylimidazole to generate PA-TMS and PA-PDMS, respectively. All three derivatization reactions were conducted separately and analyzed by EI-GC-MS and LC-HRMS. It was found that PIC, the product resulting from the carbamoylation of PA, can be detected by both EI-GC-MS and LC-HRMS, while at this concentration, the PA-TMS derivative cannot be detected by either method and the PA-PDMS derivative can only be detected by EI-GC-MS. It is important to note that the PA-TMS derivative is particularly challenging to detect when the PA is present in low concentrations and when it needs to be generated in situ (i.e., derivatization of a matrix containing PA and not spiking a standard of PA-TMS in pure form in a matrix as described in our stability studies). Given the standard conditions for the trimethylsilylation (65 °C, 3 h), it is likely that PA-TMS experiences rapid degradation, an event in which it is further accelerated under the acidic conditions used for LC-HRMS analysis. With regard to the second silylation derivative, PA-PDMS, its detection by EI-GC-MS is expected as this is a methodology that was specifically developed to produce a more stable PA silyl derivative and under milder reaction conditions.^34^ However, it appears that the PA-PDMS derivative does not survive the acidic conditions used during the LC-HRMS analysis. No trace of PA-PDMS was detected in our studies. All of the data associated with these comparative studies are provided in the Supporting Information (pages S20–S25), and they highlight the importance of strategically adding the carbamoylation of PA as a first line derivatization method for this important nerve agent marker during OPCW PTs and other routine analyses.

## 3 Conclusions

In summary, a new method for the analysis of pinacolyl alcohol by EI-GC-MS and LC-HRMS has been introduced. As PA features a low molecular weight (*m*/*z* = 102) and early eluting time during chromatographic analyses, it is a particularly challenging analyte to detect and correctly identify when present at low concentrations in a matrix. In this work, 1,1′-carbonyldimidazole (CDI) was used to modify PA in two different soil matrices at concentrations relevant to chemical weapons convention (CWC) standards. Reaction between PA and CDI yields pinacolyl imidazolyl carbamate (PIC) which is a carbamate product with an enhanced chromatographic profile for EI-GC-MS analysis as well as detectability by LC-HRMS. PA, when spiked at 1 and 10 μg/g separately in each soil matrix, was effectively converted to PIC and detected as such using EI-GC-MS and LC-HRMS. The protocol is composed of two parts: an extraction step followed by derivatization of the extracted PA. For the VA soil, DCM was found to be the most optimal solvent for the extraction of PA for both spiked concentrations, while 1-CB was found to work best for its extraction from silt sediment. However, another efficient solvent for the extraction of PA from both soil matrices was found to be CHCl_3_. Therefore, CHCl_3_ can be used interchangeably with DCM or 1-CB when dealing with the analysis of PA in soil matrices. After the evaporation of each extract to yield a residue, the derivatization was carried out by redissolving it in DCM, treating it with CDI and heating the mixture to 55 °C for 3 h. Although, it is suggested that the reaction mixture could be concentrated as much as possible when conducting samples with very low analyte concentrations, in our case direct analysis of this mixture by EI-GC-MS was successful at detecting PIC with 3 h representing the optimal time for all the PA to be converted to PIC. In parallel, the same samples were analyzed by LC-HRMS and MS-MS with no need for solvent exchange, yielding similar results with unequivocal detection of PIC, providing a protocol that yields samples that can be employed to yield analysis by two orthogonal analytical techniques. The results herein highlight the efficiency of CDI as a derivatizing agent for the analysis and detection of PA in soil matrices collected by CWC inspection teams conducting forensic analysis in settings of alleged chemical weapons use.

## 4 Materials and Methods

### 4.1 Chemicals, Reagents, and Equipment

All chemicals were purchased from commercial suppliers and used as received. Pinacolyl alcohol (3,3-dimethyl-2-butanol, PA), 1,1′-carbonyldimiidazole (CDI), dichloromethane, 1-chlorobutane, chloroform-*d*_3_, chloroform, ethyl acetate, methanol, acetone, iodine, and clean sandy loam soil were purchased from Sigma-Aldrich (St. Louis, MO, USA). Sodium carbonate, anhydrous sodium sulfate, and acetonitrile were purchased from Acros Organics (Westchester, PA, USA). Autosampler vials and glass inserts were purchased from Agilent Technologies (Santa Clara, CA, USA). Acrodisc PTFE syringe filters (0.45 μm) were purchased from Pall laboratories (Port Washington, NY, USA). The standard PIC was synthesized in house. Virginia Type A soil and silt sediment were obtained from the Forensic Science Center (FSC) soil sample collection. For the synthesis of the standards, thin layer chromatography (TLC) was used to monitor the course of the acylations using Agela Technologies glass back MF_254_ TLC plates (pH ∼ 5), and detection of products as well as intermediates was accomplished with UV light (λ = 254 nm) in conjunction with development of color with ceric ammonium molybdate (CAM)^51^ and iodine vapor.^52,53^ Wheaton glass scintillation vials (20 mL capacity), glass vials (4 mL capacity), and 40 mL amber glass vials were purchased from VWR (Radnor, PA, USA). Purifications were done by flash column chromatography using a Biotage Isolera purification system using Biotage Sfär silica high capacity duo cartridges (10 G) using a gradient of DCM → 10% MeOH/DCM over 4 column volumes.

### 4.2 EI-GC-MS Analysis

A 6890 Agilent GC with 5975 MS detector equipped with a split/splitless injector was used for EI-GC-MS analyses.^54,55^ The GC column used for the analysis was an Agilent DB-5MS UI capillary column (30 m × 0.25 mm × 0.25 μm). Ultrahigh purity helium was used as the carrier gas at 0.8 mL/min. The injector temperature was 250 °C, while the injection volume was 1 μL. The oven temperature program was as follows: 40 °C, held for 3 min, increased at 8 °C/min to 300 °C, held for 3 min. The MS ion source and quadrupole temperatures were 230 and 150 °C, respectively. Electron ionization was used with ionization energy of 70 eV. The MS was operated to scan from *m*/*z* = 29 to *m*/*z* = 600 in 0.4 s.

### 4.3 LC-HRMS Analysis

LC-HRMS analysis was conducted in a Thermo Scientific Vanquish Flex HPLC with a Thermo Scientific Q Exactive HF-X.^56^ The LC column used for the analysis was a Waters Acquity HSS T3, 1.8 μm. Ultrahigh purity nitrogen served as the collision gas. Mobile phases used were A = water/0.1% formic acid and B = acetonitrile/0.1% formic acid. The solvent gradient was 25 min long as it was as follows: initial 1%, hold for 2 min, then linear ramp to 10% B over 6 min; ramp to 95% B over 7 min, hold at 95% B for 2 min, and re-equilibrate at 1% B for 8 min. Samples (volume size = 10 μL) were injected into LC-HRMS for analysis. The LC column temperature was set at 40 °C, and the MS was operated to scan from *m*/*z* = 75 to 750. MS acquisition was performed on a Thermo Scientific Q Exactive HF-X mass spectrometer operated using heated electrospray ionization (HESI) in positive ion mode with high resolution accurate mass to ≤3 ppm. The MS experiment is composed of a full MS spectrum (*m*/*z* = 75–750) at a resolving power setting of 30000 (full width at half-maximum (fwhm) at *m*/*z* = 200) followed by data dependent MS–MS with normalized higher energy collisional dissociation (HCD) energies of 30% at resolving power of 30000 (fwhm at *m*/*z* = 200).

### 4.4 Preparation of PA-Spiked Soil Matrices

A stock PA solution (1 mg/mL, 1000 ppm) in DCM was prepared and used for the preparation of the stock solutions (1 and 0.1 ppm) used for the spiking of both soil matrices (i.e., VA soil and silt sediment). The 1 ppm solution was prepared by taking 3 mL of the 1000 ppm stock solution and diluting it to 30 mL with DCM in a 40 mL glass amber vial. The 0.1 ppm solution was prepared by taking 1 mL of the 1 ppm stock solution and diluting to 10 mL with DCM. For the preparation of the 10 ppm spiked soil, VA soil or silt sediment (300 mg) was treated with 3 mL of the 1 ppm PA solution while the 1 ppm spiked soil was prepared by treating each matrix (300 mg) with 3 mL of the 0.1 ppm PA solution. Both soil suspensions in DCM were evaporated carefully using a rotary evaporator or a low stream of nitrogen.

### 4.5 Extraction and Derivatization of PA from Virginia Type A Soil and Silt Sediment

VA soil (300 mg) containing the PA in both concentrations (1 and 10 μg/g) was extracted with DCM or CHCl_3_ (3 × 2 mL), the collected extracts were syringe-filtered through a 0.45 μm Acrodisc filter, and the solvent gently evaporated using a nitrogen stream to dryness. The evaporation yielded a dark brown residue that was reconstituted in DCM (500 μL), placed in a 2 mL autosampler vial equipped with a micro stir bar and treated with CDI (10 mg). The resulting mixture was heated to 55 °C for 3 h, cooled to ambient temperature, and directly analyzed by EI-GC-MS and LC-HRMS. In similar fashion, silt sediment (300 mg) containing the PA in both concentrations (1 and 10 μg/g), was extracted with 1-CB or CHCl_3_ (3 × 2 mL), the collected extracts were syringe-filtered through a 0.45 μm Acrodisc filter and the solvent gently evaporated using a nitrogen stream to dryness. The evaporation yielded a dark yellow residue that was reconstituted in DCM (500 μL), placed in a 2 mL autosampler vial equipped with a micro stir bar, and treated with CDI (10 mg). The resulting mixture was heated to 55 °C for 3 h, cooled to ambient temperature and directly analyzed by EI-GC-MS and LC-HRMS.

## Supplementary Material



## Notes

EI-GC-MS stability studies on PIC; CDI derivatization application and analysis by EI-GC-MS; nuclear magnetic resonance data on PIC; EI-GC-MS and statistical data on PA extraction efficiencies; determination of LOD/LOQ values; comparative studies between carbamoylation and silylation methods (PDF)
