# Supplementary material for: Carbamoylation as an Effective Tool in the Analysis of the Soman Nerve Agent Marker Pinacolyl Alcohol in Soil Matrices by EI-GC-MS and LC-HRMS
Source: ACS Omega. 2026 May 1;11(18):26931–41. doi: 10.1021/acsomega.6c00271 (PMC13177215; doi:10.1021/acsomega.6c00271)
Supplement: Supplementary file 1 [file ao6c00271_si_001.pdf]

-Supporting Information-

"Carbamoylation as an Effective Tool in the Analysis of the Soman Nerve Agent Marker Pinacolyl Alcohol in Soil Matrices by EI-GC-MS and LC-HRMS"

David Baliu-Rodriguez[1,2], David S. Cho[2-4], Adele Panasci-Nott[2-4], Saphon Hok[2-4], Alexander K. Vu[2-4], Mark L. Dreyer[2-4], Carlos A. Valdez[1-4,*]

[1]Biosciences and Biotechnology Division, [2]Physical and Life Sciences Directorate, [3]Forensic Science Center, [4]Global Security Directorate, Lawrence Livermore National Laboratory, Livermore, CA, 94550, USA.

Table of Contents

Content	Page
Conversion of PA into PIC using CDI	S2
Stability of PIC	S3
Thermal stability of PIC	S3
Thermal stability of PIC (Figures S4-S11)	S4
Derivatization of isomeric alcohols with CDI	S7
Figures S12, S13	S7
Figures S14-S16	S8
Figures S17-S19	S9
Nuclear Magnetic Resonance Spectra for PIC	S10
Table S1. Extraction efficiencies for PA from VA soil (1 μg/g)	S12
Table S2. Extraction efficiencies for PA from VA soil (10 μg/g)	S12
Table S3. Extraction efficiencies for PA from silt (1 μg/g)	S12
Table S4. Extraction efficiencies for PA from silt (10 μg/g)	S12
Statistical analysis on extraction recoveries for PA from matrices	S14
LOD and LOQ values determination for PIC in both soils	S20
Comparative studies between silylation and carbamoylation	S22
GC-MS comparative studies results	S23
LC-HRMS comparative studies results	S25

Conversion of PA into PIC (time study)

Derivatization of PA using CDI to produce PIC is outlined below:

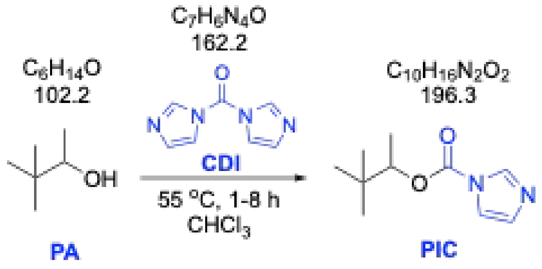

As PA is not detectable by LC-MS, the extent of the CDI derivatization by EI-GC-MS. The experimental setup was as follows: PA at a 25 µg/mL in $CHCl_3$ extracts of both soils (VA and silt) were treated with CDI (10-fold excess) in autosampler vials and the solutions were heated to 55 °C over 8 hours. Aliquots from each soil extract, after cooling the mixture to ambient temperature (~23-24 °C) were taken at timepoints: 1, 2, 3, 5 and 8 hours and analyzed by EI-GC-MS. In the graphs below, the orange bars represent the concentration of pinacolyl alcohol, while the blue ones represent the product PIC accumulating from the reaction between PA and CDI. The results of these experiments are shown in Figures S1 and S2 below. From the data collected, it can be concluded that the reaction is completed within 2 hours with the extended heating up to 3 hours serving to ensure that all the PA has been successfully converted to the PIC derivative.

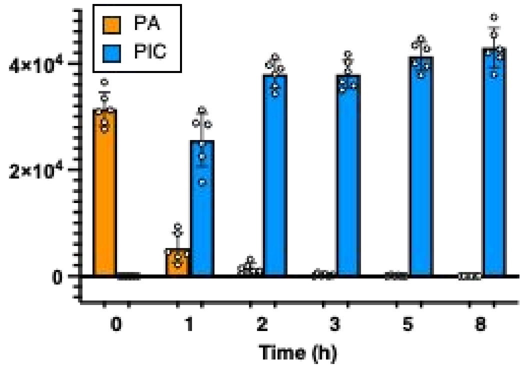

Figure S1. Timeline for the conversion of PA into PIC by CDI at 55 °C for the Virginia type A (VA) soil extract. Average (n=6) peak areas (+/- the standard deviation).

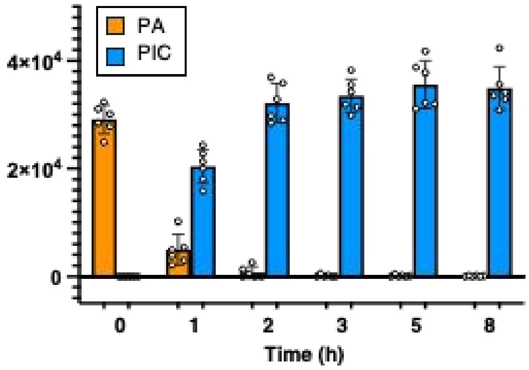

Figure S2. Timeline for the conversion of PA into PIC by CDI at 55 °C for the silt sediment extract. Average (n=6) peak areas (+/- the standard deviation).

Stability of PIC (EI-GC-MS studies)

The effect of residual moisture after solvent evaporation on the PIC product was setup as follows: 5 solutions of PIC at a 25 µg/mL concentration in DCM (5 x 100 µL) were prepared and evaporated using a nitrogen stream and the vials left open for 2, 4, 8, 24, 48, 72 and 96 h in the fume hood (humidity level measured at 82%). At the end of each time, each residue was redissolved in DCM (100 µL) and analyzed by EI-GC-MS. All analyses were compared to a standard PIC solution (@ 25 µg/mL in DCM) to evaluate any hydrolysis of the derivative. In parallel and for comparison purposes, the PA-TMS (pinacolyl alcohol trimethylsilyl) derivative was prepared to create a point of comparison for the stability of both derivatives. Again, 5 solutions of PA-TMS at a 25 µg/mL concentration in DCM (5 x 100 µL) were prepared and evaporated using a nitrogen stream and the vials left open for 2, 4, 8, 24, 48, 72 and 96 h in the fume hood. At the end of each time, each residue was redissolved in DCM (100 µL) and analyzed by EI-GC-MS. **Figure S3** below shows the results and from this data it can be concluded that the carbamate-derived PA exhibits superior stability than its silylated counterpart over 72 hours at ambient temperature.

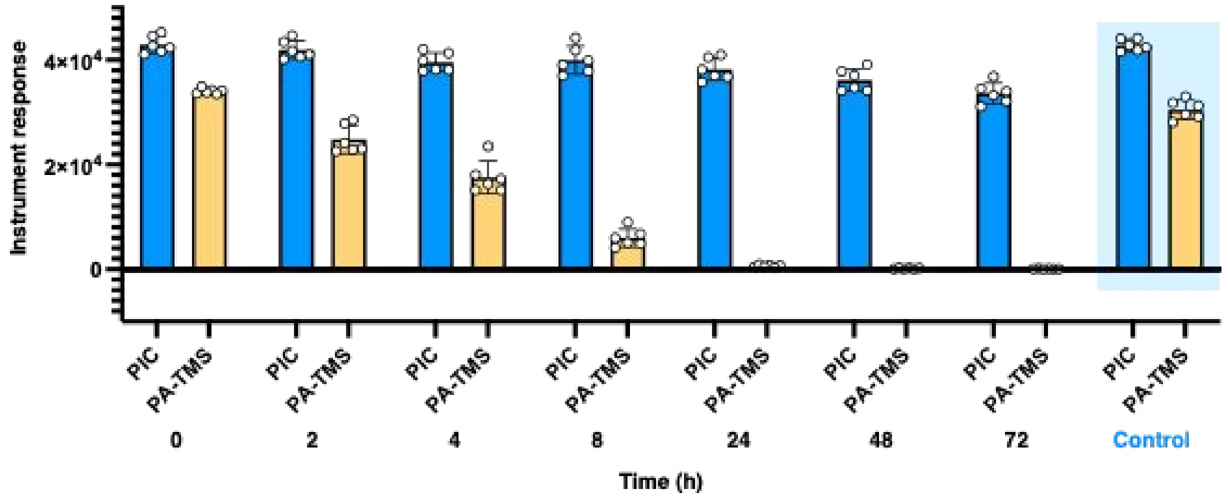

Figure S3. Stability of PIC (blue bar) and PA-TMS (yellow bar) towards hydrolysis (humidity, 82%) over a 72 hour period. Last set of bars represent the control experiment which is the signal intensity of a 25 µg/mL solutions of PIC and PA-TMS. Average (n=6) peak areas (+/- the standard deviation).

Thermal stability of PIC

The thermal stability of PIC in the GC port was investigated. A possible breakdown of PIC in the GC port would occur according to the following equation:

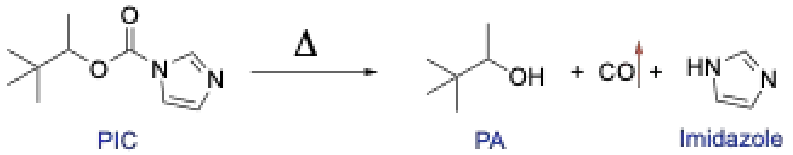

Therefore, PIC would undergo decomposition with the extrusion of carbon monoxide (CO) to yield pinacolyl alcohol (PA) and imidazole. In order to assess the stability of PIC in the GC port and overall GC conditions used, a pure standard of PIC was prepared in DCM at two separate concentrations (10 and 100 μg/mL). Each solution was injected (n=3) and analyzed in triplicate by EI-GC-MS. **Figures S4-S11** show the GC Total Ion Chromatograms (TICs) for each run, as well as the analysis of each using the single ion extraction (SIE) mode using the most abundant MS peaks for **PA (m/z = 57), imidazole (m/z = 68) and PIC (m/z = 85)**. For the SIE analysis, the areas where PA, imidazole and the PIC product are enlarged for comparative purposes. From the SIE analysis, one can see that the PIC product is pure and that no signals arising in the TIC and extracted ion chromatograms show any PA or imidazole. These experiments show that PIC is thermally stable under the GC conditions used for its analysis.

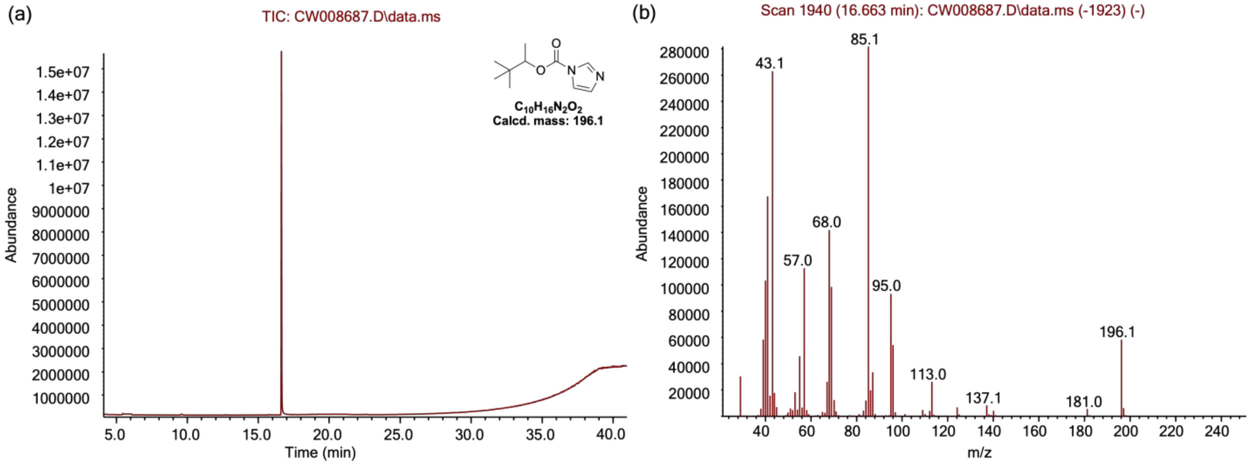

Figure S4. (a) TIC for pure PIC injected at a 10 μg/mL concentration in DCM and (b) mass spectrum for the material at this concentration.

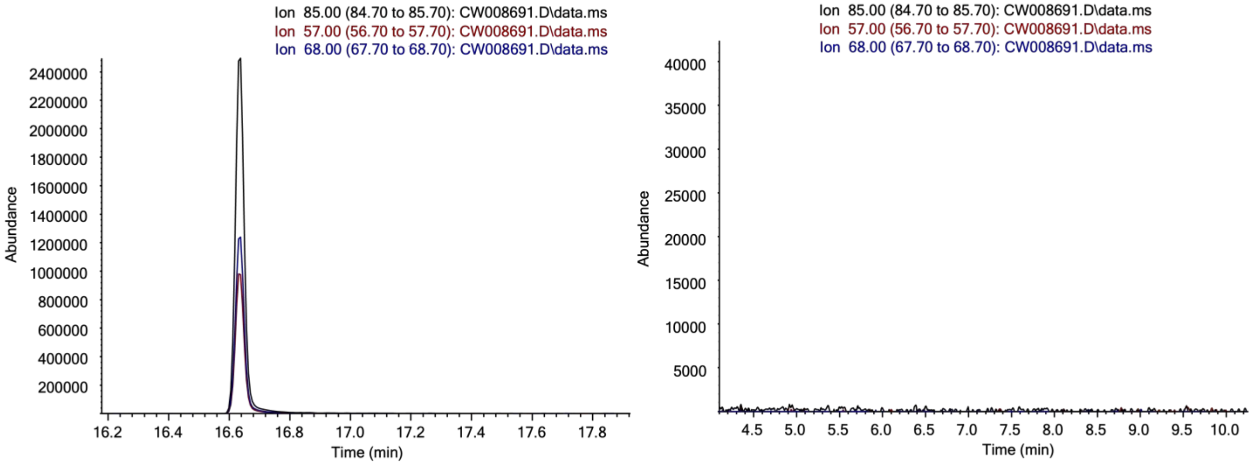

Figure S5. Selected ion extraction analysis of pure PIC using m/z = 85, 57 and 68 as base peak ions for PIC, PA and imidazole respectively (first replicate of triplicate measurements). The PA and imidazole elute with retention times of RT ~ 4-5 min and RT = 8.1-8.3 min respectively.

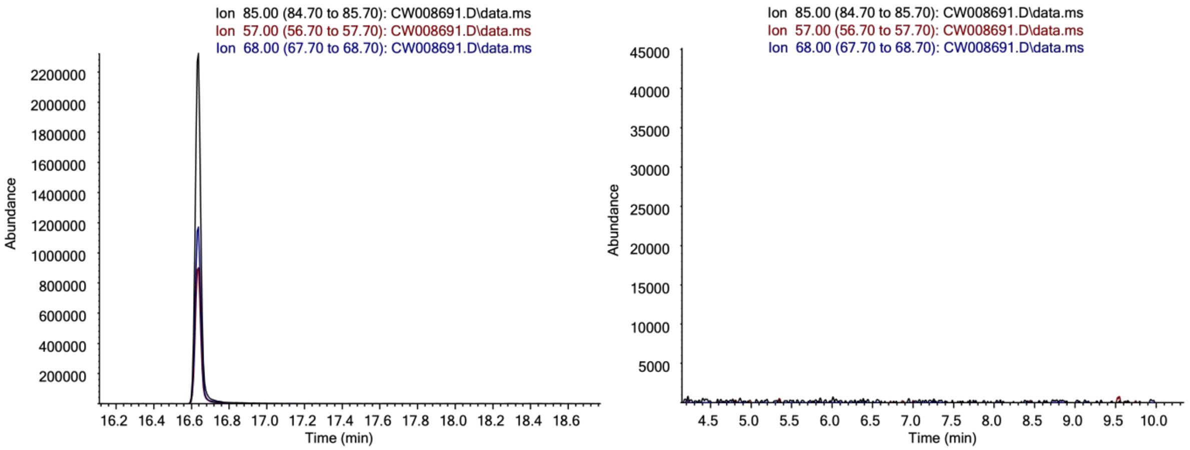

Figure S6. Selected ion extraction analysis of pure PIC using m/z = 85, 57 and 68 as base peak ions for PIC, PA and imidazole respectively (<u>second</u> replicate of triplicate measurements). The PA and imidazole elute with retention times of RT ~ 4-5 min and RT = 8.1-8.3 min respectively.

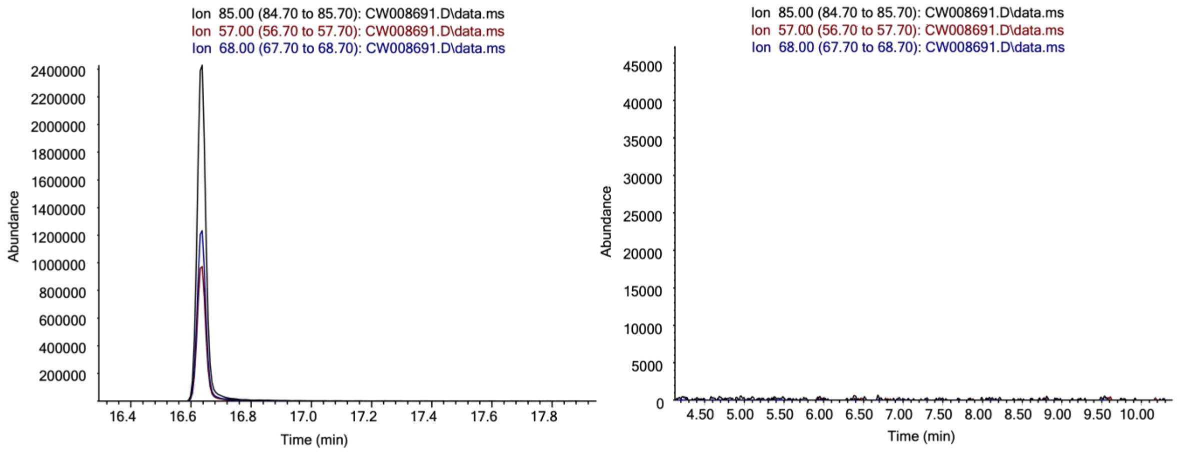

Figure S7. Selected ion extraction analysis of pure PIC using m/z = 85, 57 and 68 as base peak ions for PIC, PA and imidazole respectively (<u>third</u> replicate of triplicate measurements). The PA and imidazole elute with retention times of RT ~ 4-5 min and RT = 8.1-8.3 min respectively.

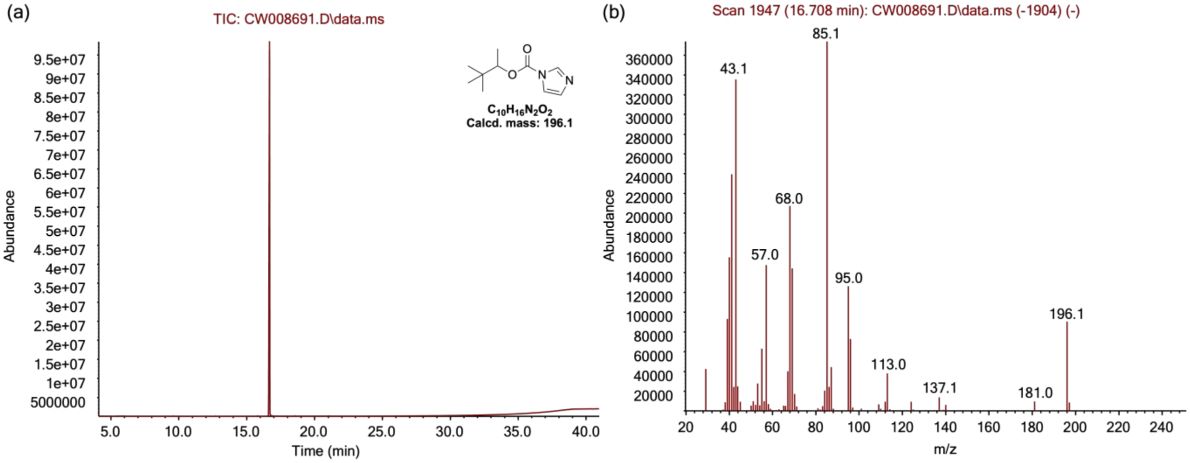

Figure S8. (a) TIC for pure PIC injected at a 100 μg/mL concentration in DCM and (b) mass spectrum for the material at this concentration.

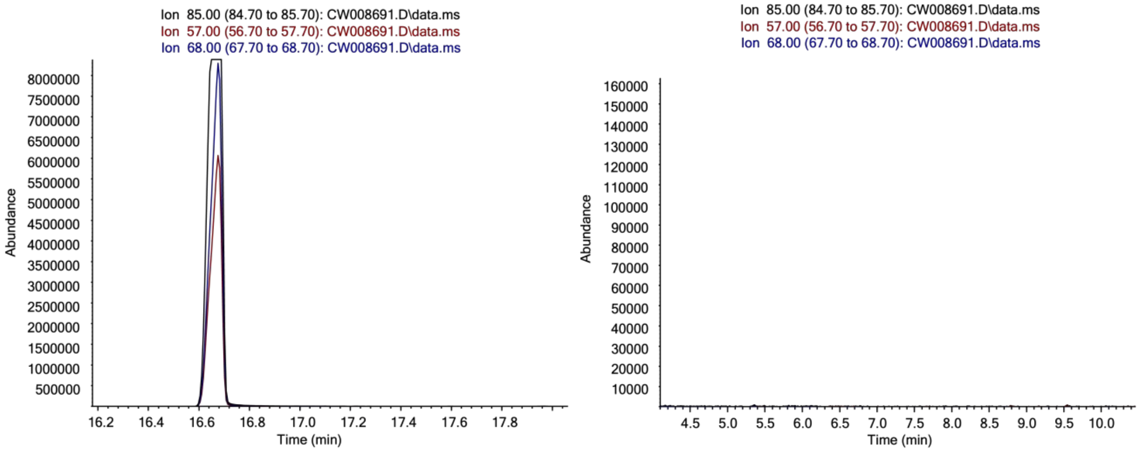

Figure S9. Selected ion extraction analysis of pure PIC using m/z = 85, 57 and 68 as base peak ions for PIC, PA and imidazole respectively (<u>first</u> replicate of triplicate measurements). The PA and imidazole elute with retention times of RT ~ 4-5 min and RT = 8.1-8.3 min respectively.

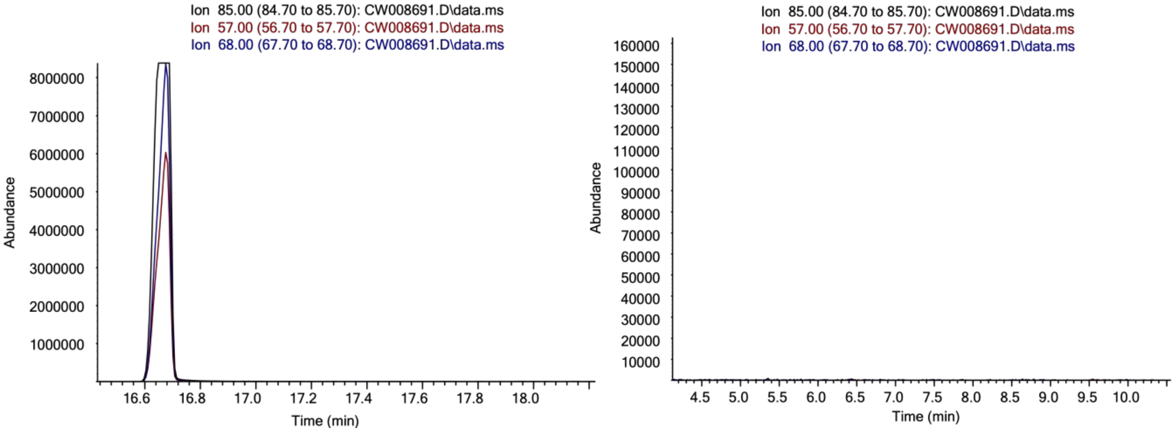

Figure S10. Selected ion extraction analysis of pure PIC using m/z = 85, 57 and 68 as base peak ions for PIC, PA and imidazole respectively (<u>second</u> replicate of triplicate measurements). The PA and imidazole elute with retention times of RT ~ 4-5 min and RT = 8.1-8.3 min respectively.

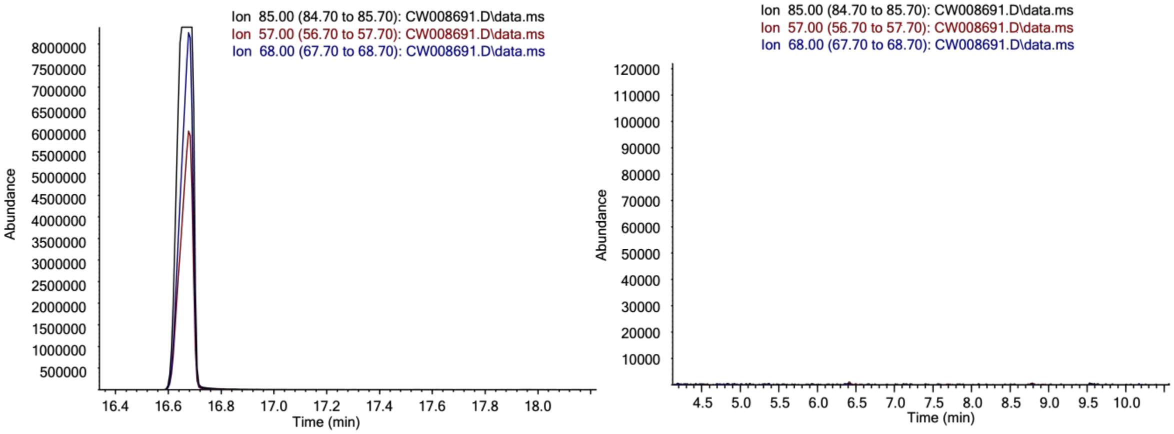

Figure S11. Selected ion extraction analysis of pure PIC using m/z = 85, 57 and 68 as base peak ions for PIC, PA and imidazole respectively (<u>third</u> replicate of triplicate measurements). The PA and imidazole elute with retention times of RT ~ 4-5 min and RT = 8.1-8.3 min respectively.

Derivatization of isomeric alcohols with CDI

The methodology described herein can be applicable to other alcohols. To this end, we chose a panel of 7 isomeric alcohols to PA and also cyclohexanol which was included for its connection to the nerve agent cyclosarin (GF).

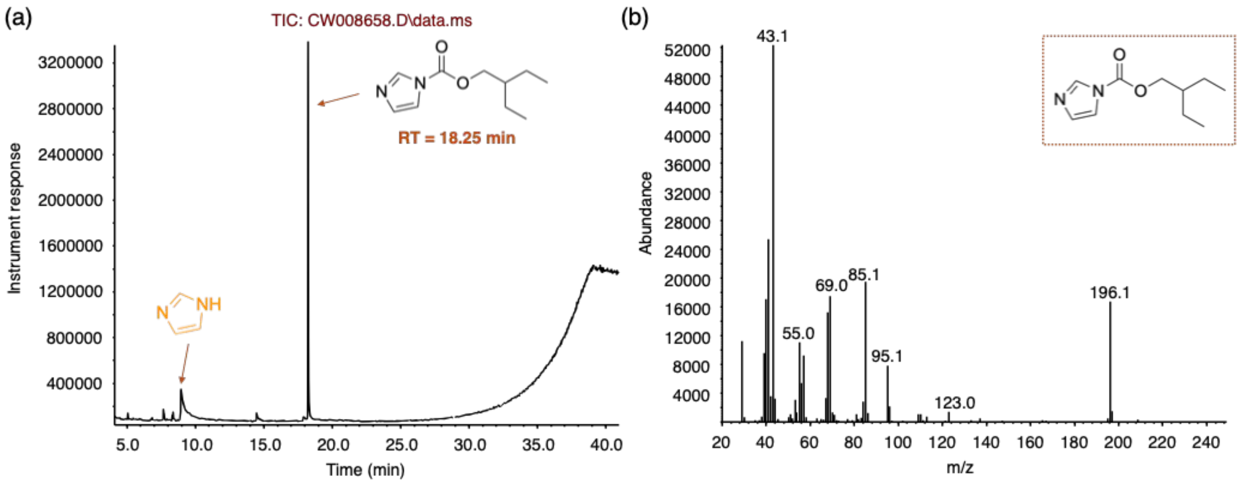

Figure S12. (a) TIC of the reaction mixture between 2-ethyl-1-butanol and CDI and (b) the mass spectrum of the imidazolyl carbamate product.

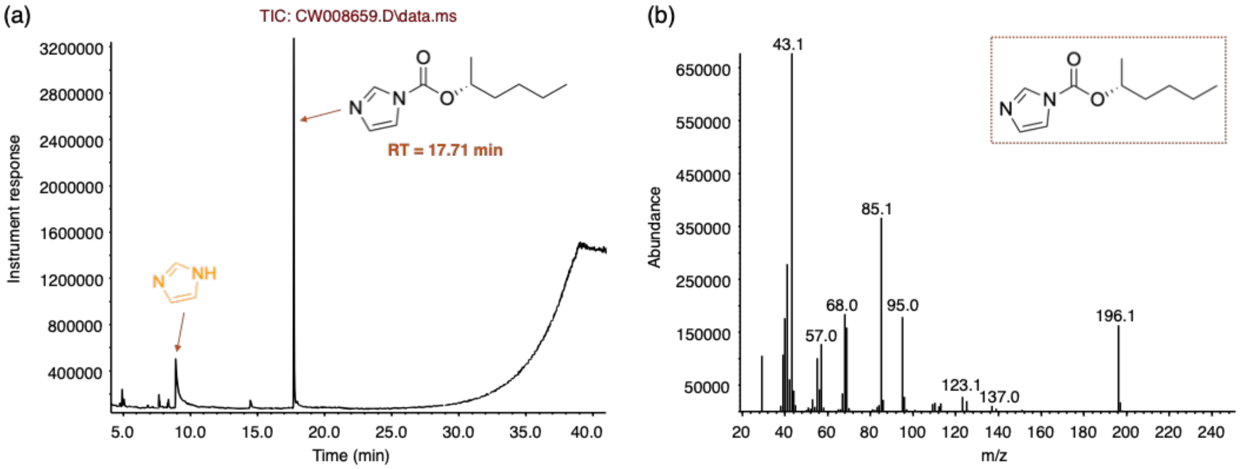

Figure S13. (a) TIC of the reaction mixture between (*R*)-2-hexanol and CDI and (b) the mass spectrum of the imidazolyl carbamate product.

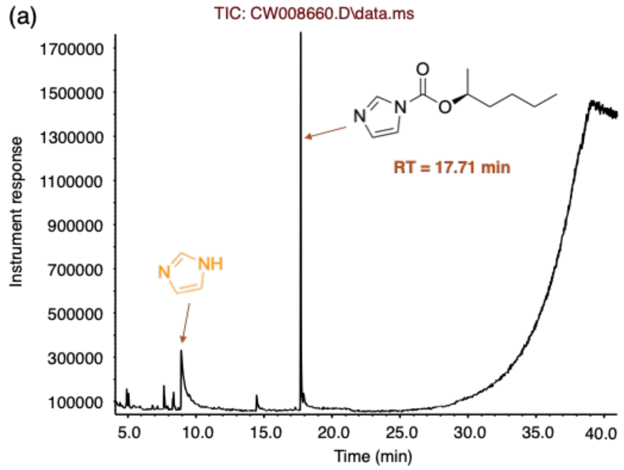

Figure S14. (a) TIC of the reaction mixture between (*S*)-2-hexanol and CDI and (b) the mass spectrum of the imidazolyl carbamate product.

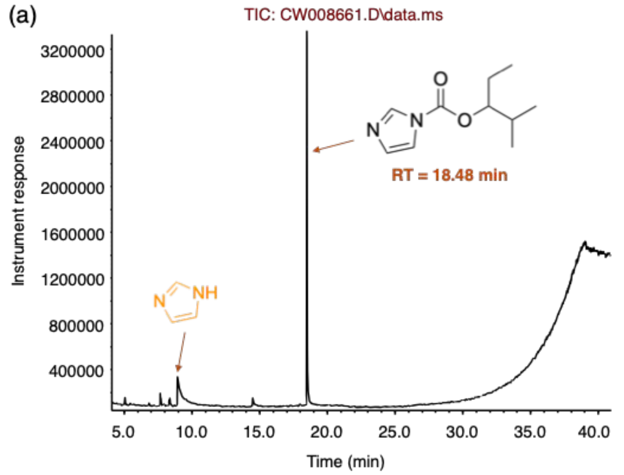

Figure S15. (a) TIC of the reaction mixture between 2-methyl-3-pentanol and CDI and (b) the mass spectrum of the imidazolyl carbamate product.

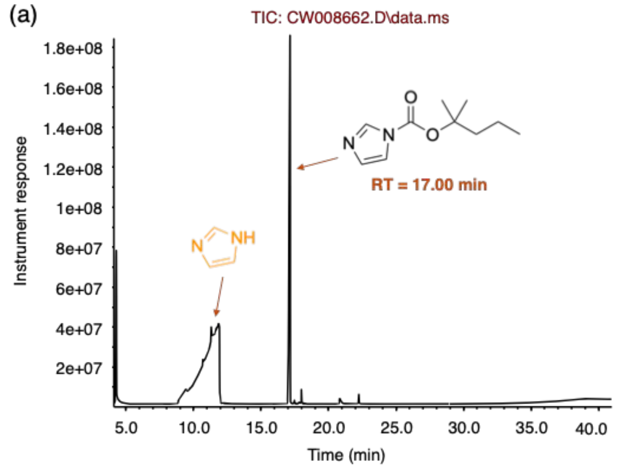

Figure S16. (a) TIC of the reaction mixture between 2-methyl-2-pentanol and CDI and (b) the mass spectrum of the imidazolyl carbamate product.

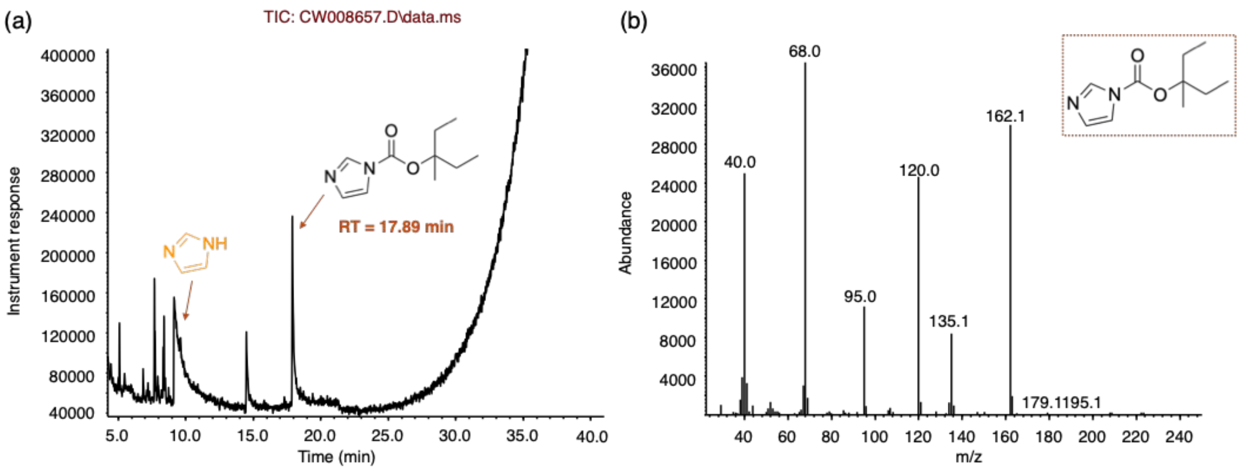

Figure S17. (a) TIC of the reaction mixture between 3-methyl-3-pentanol and CDI and (b) the mass spectrum of the imidazolyl carbamate product.

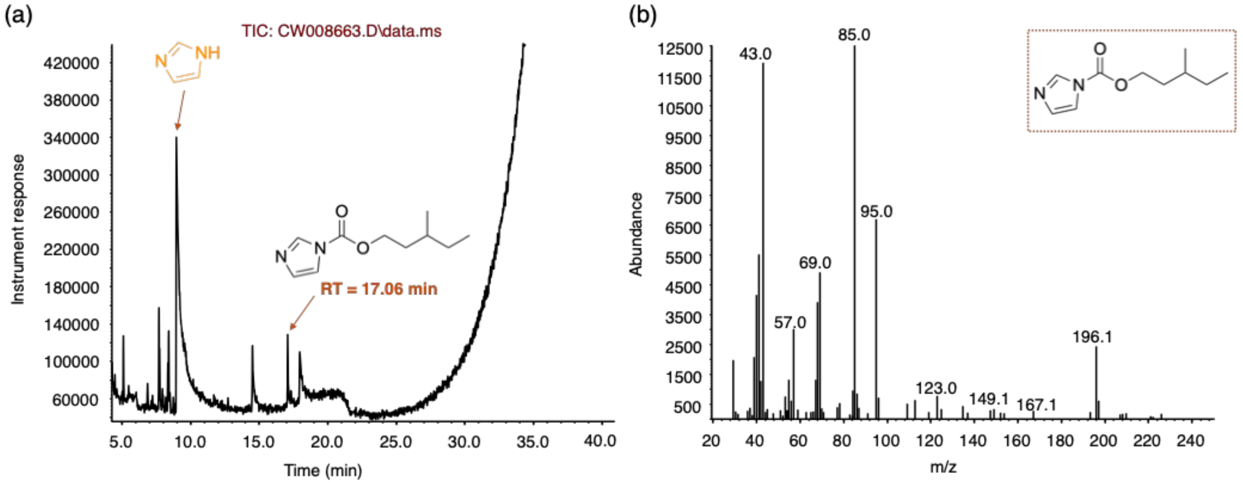

Figure S18. (a) TIC of the reaction mixture between 3-methyl-1-pentanol and CDI and (b) the mass spectrum of the imidazolyl carbamate product.

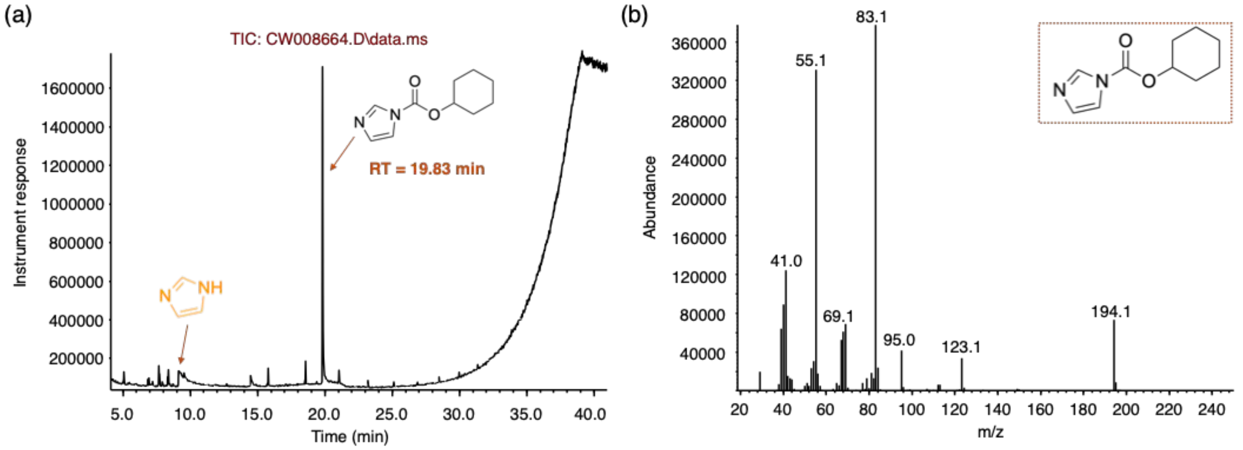

Figure S19. (a) TIC of the reaction mixture between cyclohexanol and CDI and (b) the mass spectrum of the imidazolyl carbamate product

Nuclear Magnetic Resonance Spectra for PIC

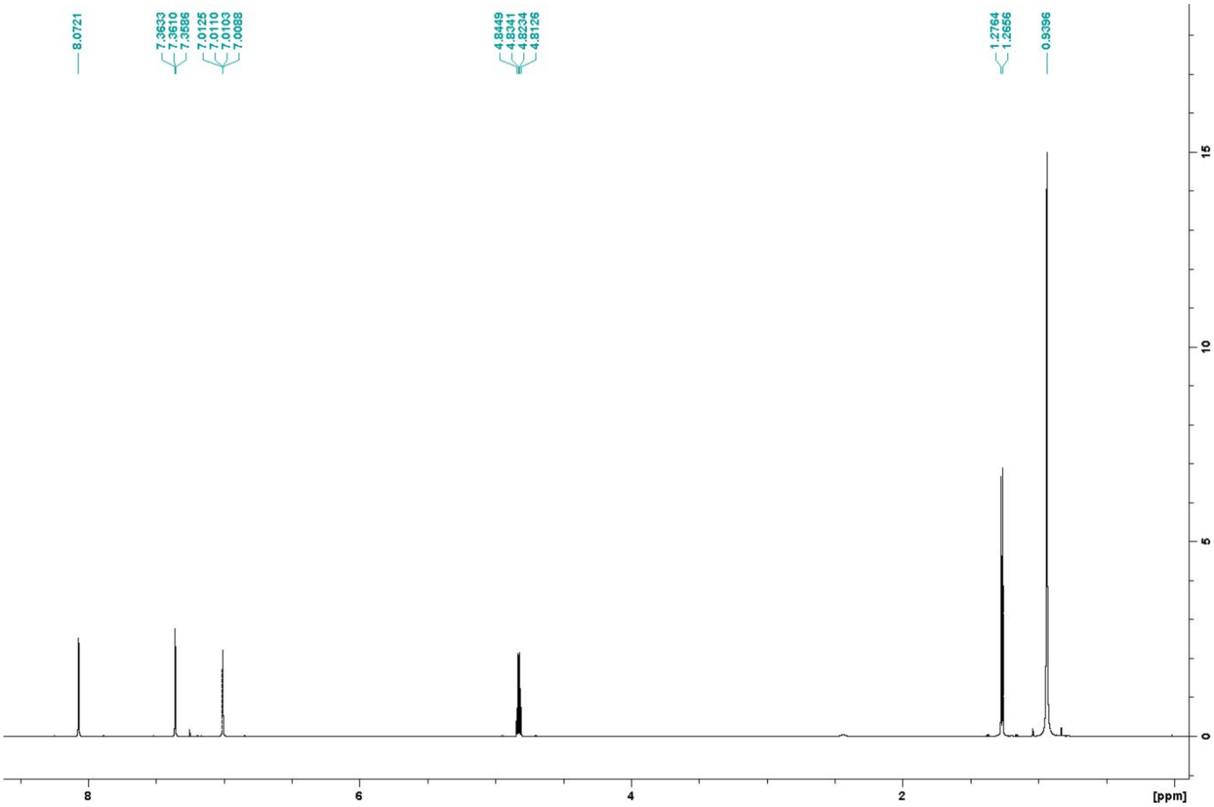

Figure S20. ^{1}H NMR spectrum of PIC (Full, $CDCl_3$, 600 MHz).

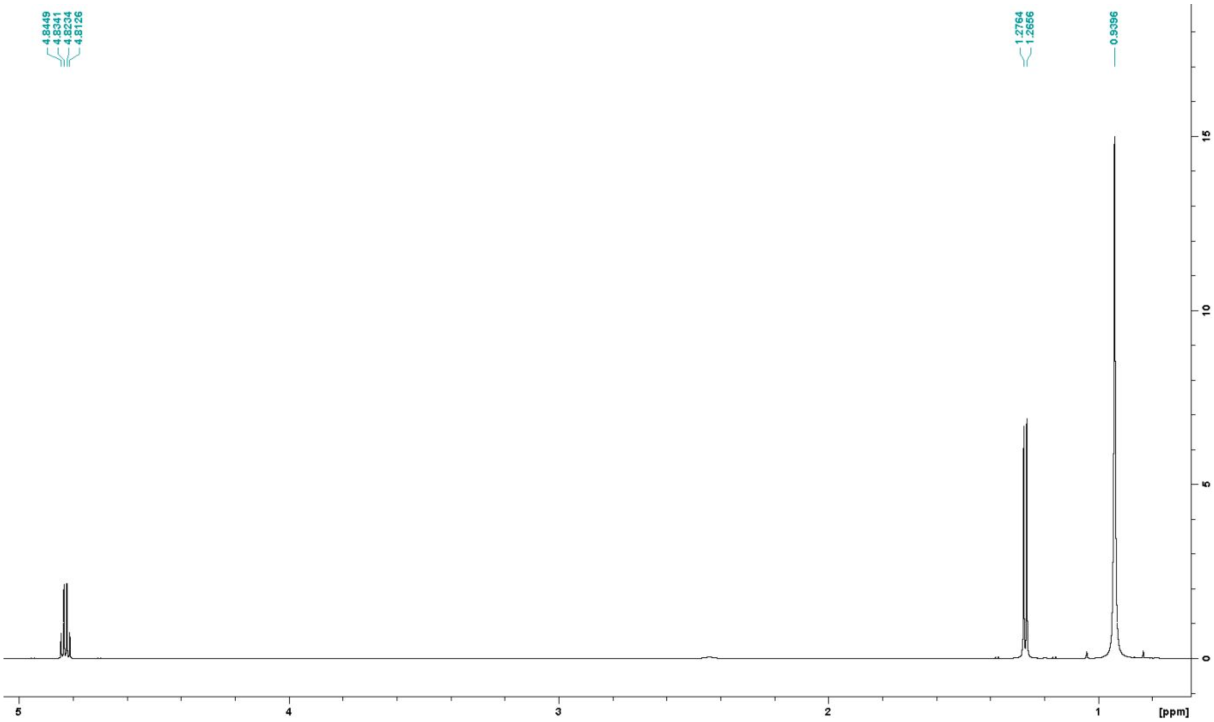

Figure S21. ^{1}H NMR spectrum of PIC (Expansion, $CDCl_3$, 600 MHz).

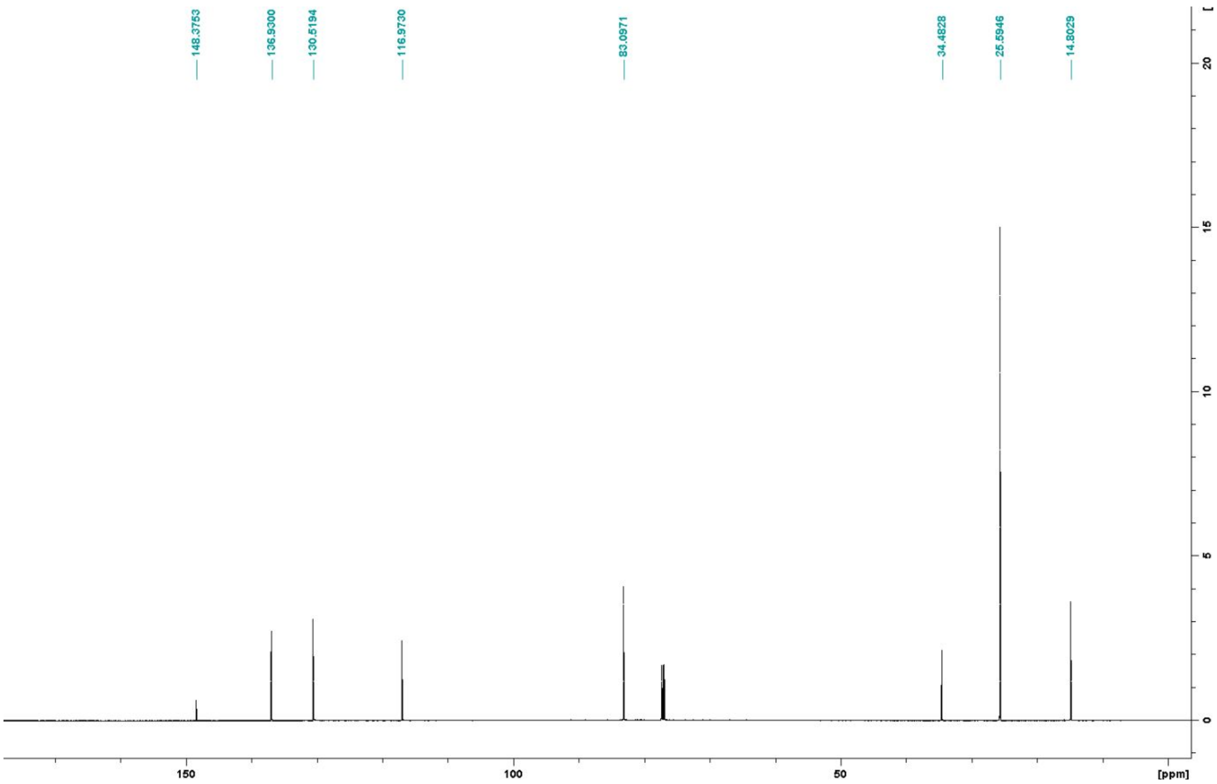

Figure S22. ^{13}C NMR spectrum of PIC ($CDCl_3$, 150 MHz).

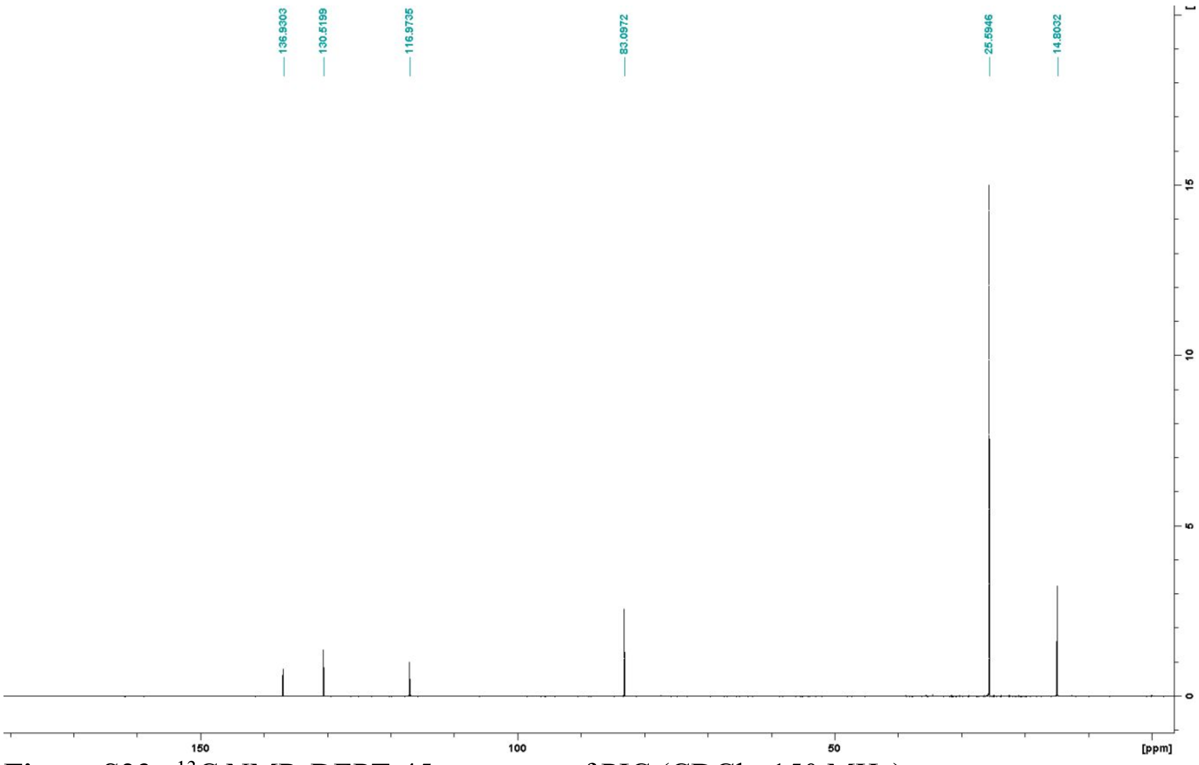

Figure S23. ^{13}C NMR-DEPT-45 spectrum of PIC ($CDCl_3$, 150 MHz).

Table S1. Extraction efficiencies for PA from VA soil when spiked at 1 µg/g separately using four solvents: dichloromethane (DCM), ethyl acetate (EtOAc), acetonitrile (ACN) and 1-chlorobutane (1-CB). Values correspond to the area under the curve of the peak generated when PA is analyzed by SIE (m/z = 85).

Solvent →	DCM	EtOAc	ACN	1-CB	Control [1 µg/g]
1	1899	801	907	1245	2201
2	1779	545	888	1333	2077
3	2011	874	656	1278	1989
4	1814	129	779	1310	2118
5	1801	339	997	1332	2204
6	1698	890	885	1145	2210

Table S2. Extraction efficiencies for PA from VA soil when spiked at 10 µg/g separately using four solvents: dichloromethane (DCM), ethyl acetate (EtOAc), acetonitrile (ACN) and 1-chlorobutane (1-CB). Values correspond to the area under the curve of the peak generated when PA is analyzed by SIE (m/z = 85).

Solvent →	DCM	EtOAc	ACN	1-CB	Control [10 µg/g]
1	20522	9567	10564	11223	27023
2	21323	8963	11231	10043	28940
3	19523	10343	8734	11002	29878
4	20342	8231	9744	12321	25567
5	19652	10823	10522	12975	23903
6	22212	9232	9034	8342	26788

Table S3. Extraction efficiencies for PA from silt when spiked at 1 µg/g separately using four solvents: dichloromethane (DCM), ethyl acetate (EtOAc), acetonitrile (ACN) and 1-chlorobutane (1-CB). Values correspond to the area under the curve of the peak generated when PA is analyzed by SIE (m/z = 85).

Solvent →	DCM	EtOAc	ACN	1-CB	Control [1 µg/g]
1	1301	801	907	1899	2201
2	1011	545	888	1779	2077
3	1289	1034	656	2011	1989
4	1287	129	779	1814	2118
5	1331	339	997	1801	2204
6	1102	890	885	1698	2210

Table S4. Extraction efficiencies for PA from silt when spiked at 10 μg/g separately using four solvents: dichloromethane (DCM), ethyl acetate (EtOAc), acetonitrile (ACN) and 1-chlorobutane (1-CB). Values correspond to the area under the curve of the peak generated when PA is analyzed by SIE (m/z = 85).

Solvent →	DCM	EtOAc	ACN	1-CB	Control [10 μg/g]
1	11585	1565	14231	22432	26565
2	11932	3451	11323	21421	27564
3	12500	3524	9454	22198	27434
4	13745	6765	9983	18975	26421
5	13113	3113	11032	19434	24989
6	12041	4812	10434	18884	27412

Statistical analysis of PA extraction recoveries from both soil matrices

Three separate experiments were carried out at three spiking levels for PA (1, 25 and 100 μg/mL) for each soil (VA and silt). The soils were extracted with $CHCl_3$ and the extraction efficiencies were evaluated for each soil. The data included contains all the statistical analysis for each extraction at each spiked concentration of PA. The statistical analysis of all extracted values at each concentration and their direct comparison to a control 1, 25 and 100 μg/mL samples are given below for the VA type A soil case:

For PA spiked at a <u>1 ppm</u> concentration in VA soil:

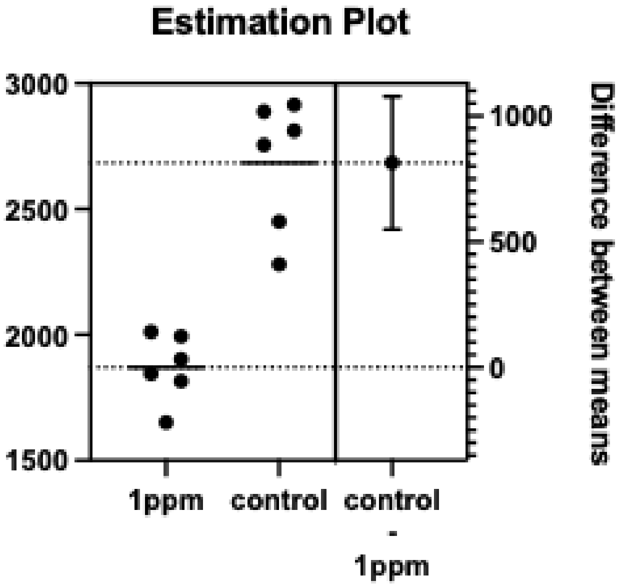

Unpaired t test	
P value	<0.0001
P value summary	****
Significantly different (P < 0.05)?	Yes
One- or two-tailed P value?	Two-tailed
t, df	t=6.868, df=10
How large is the effect?	
Mean of group A	1870
Mean of group B	2684
Difference between means (B - A) ± SEM	813.5 ± 118.5
95% confidence interval	549.6 to 1077
R squared (eta squared)	0.8251
F test to compare variances	
F, DFn, Dfd	3.800, 5, 5
P value	0.1692
P value summary	ns
Significantly different (P < 0.05)?	No
Data analyzed	
Sample size, group A	6
Sample size, group B	6

Number of values	6	6
Minimum	1652	2281
Maximum	2012	2915
Range	360.0	634.0
Mean	1870	2684
Std. Deviation	132.4	258.2
Std. Error of Mean	54.07	105.4

For PA spiked at a <u>25 ppm</u> concentration in VA soil:

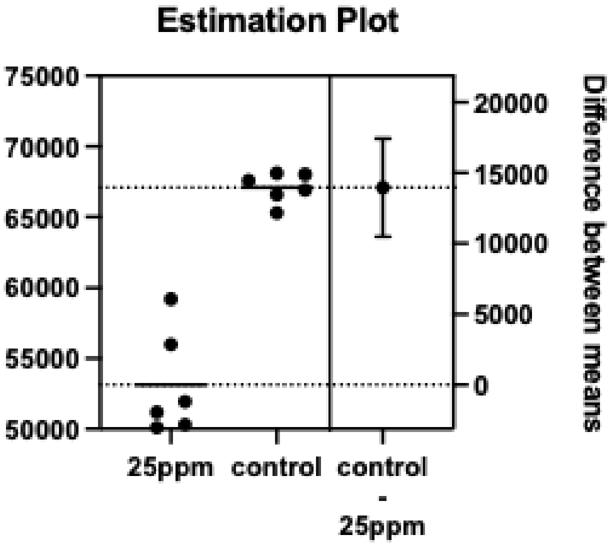

Unpaired t test	
P value	<0.0001
P value summary	****
Significantly different (P < 0.05)?	Yes
One- or two-tailed P value?	Two-tailed
t, df	t=8.970, df=10
How large is the effect?	
Mean of group A	53132
Mean of group B	67091
Difference between means (B - A) ± SEM	13959 ± 1556
95% confidence interval	10492 to 17427
R squared (eta squared)	0.8895
F test to compare variances	
F, DFn, Dfd	11.82, 5, 5
P value	0.0170
P value summary	*
Significantly different (P < 0.05)?	Yes
Data analyzed	
Sample size, group A	6
Sample size, group B	6

Number of values	6	6
Minimum	50123	65298
Maximum	59201	68121
Range	9078	2823
Mean	53132	67091
Std. Deviation	3660	1065
Std. Error of Mean	1494	434.7

For PA spiked at a 100 ppm concentration in VA soil:

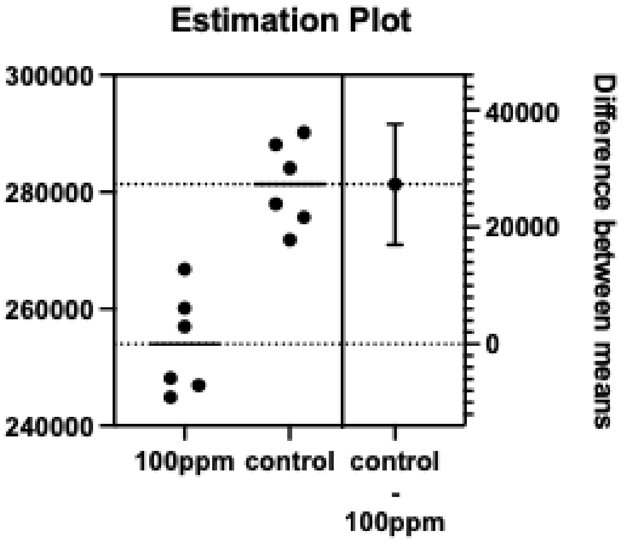

Unpaired t test	
P value	0.0001
P value summary	***
Significantly different (P < 0.05)?	Yes
One- or two-tailed P value?	Two-tailed
t, df	t=5.906, df=10
How large is the effect?	
Mean of group A	253973
Mean of group B	281307
Difference between means (B - A) ± SEM	27334 ± 4628
95% confidence interval	17022 to 37646
R squared (eta squared)	0.7772
F test to compare variances	
F, DFn, Dfd	1.426, 5, 5
P value	0.7063
P value summary	ns
Significantly different (P < 0.05)?	No
Data analyzed	
Sample size, group A	6
Sample size, group B	6

Number of values	6	6
Minimum	244875	271842
Maximum	266789	290151
Range	21914	18309
Mean	253973	281307
Std. Deviation	8692	7278
Std. Error of Mean	3548	2971

The statistical analysis of all extracted values at each concentration and their direct comparison to a standard 1, 25 and 100 µg/mL control samples are given below for the silt sediment case:

For PA spiked at a <u>1 ppm</u> concentration in silt sediment:

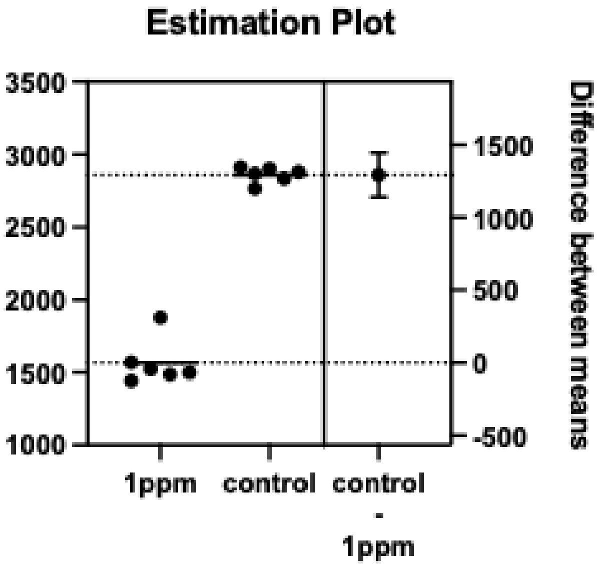

Unpaired t test	
P value	<0.0001
P value summary	****
Significantly different (P < 0.05)?	Yes
One- or two-tailed P value?	Two-tailed
t, df	t=18.96, df=10
How large is the effect?	
Mean of group A	1566
Mean of group B	2860
Difference between means (B - A) ± SEM	1294 ± 68.24
95% confidence interval	1142 to 1446
R squared (eta squared)	0.9729
F test to compare variances	
F, DFn, Dfd	8.807, 5, 5
P value	0.0322
P value summary	*
Significantly different (P < 0.05)?	Yes
Data analyzed	
Sample size, group A	6
Sample size, group B	6

Number of values	6	6
Minimum	1442	2766
Maximum	1878	2911
Range	436.0	145.0
Mean	1566	2860
Std. Deviation	158.4	53.37
Std. Error of Mean	64.66	21.79

For PA spiked at a <u>25 ppm</u> concentration in silt sediment:

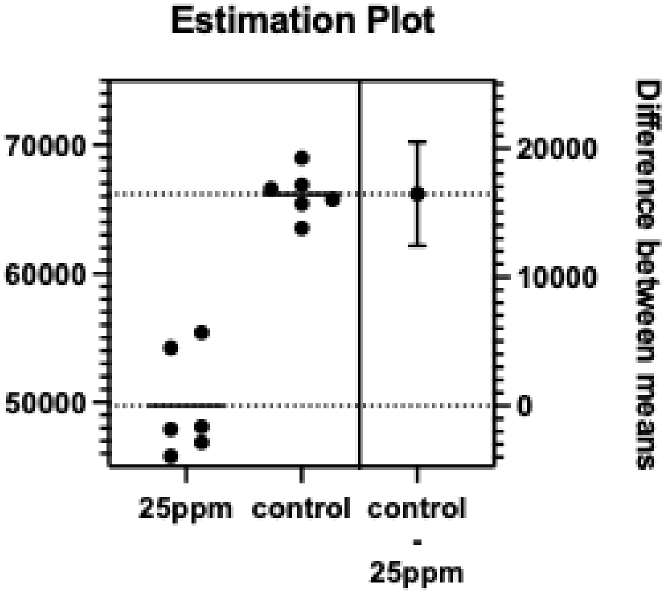

Unpaired t test	
P value	<0.0001
P value summary	****
Significantly different (P < 0.05)?	Yes
One- or two-tailed P value?	Two-tailed
t, df	t=9.097, df=10
How large is the effect?	
Mean of group A	49731
Mean of group B	66195
Difference between means (B - A) ± SEM	16464 ± 1810
95% confidence interval	12432 to 20497
R squared (eta squared)	0.8922
F test to compare variances	
F, DFn, Dfd	5.050, 5, 5
P value	0.1000
P value summary	ns
Significantly different (P < 0.05)?	No
Data analyzed	
Sample size, group A	6
Sample size, group B	6

Number of values	6	6
Minimum	45821	63530
Maximum	55424	68978
Range	9603	5448
Mean	49731	66195
Std. Deviation	4050	1802
Std. Error of Mean	1653	735.8

For PA spiked at a <u>100 ppm</u> concentration in silt sediment:

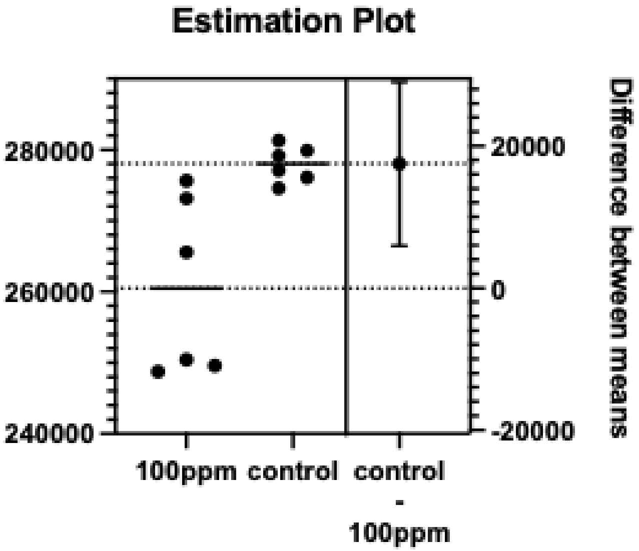

Unpaired t test	
P value	0.0070
P value summary	**
Significantly different (P < 0.05)?	Yes
One- or two-tailed P value?	Two-tailed
t, df	t=3.380, df=10
How large is the effect?	
Mean of group A	260530
Mean of group B	278023
Difference between means (B - A) ± SEM	17494 ± 5176
95% confidence interval	5960 to 29028
R squared (eta squared)	0.5332
F test to compare variances	
F, DFn, Dfd	24.11, 5, 5
P value	0.0033
P value summary	**
Significantly different (P < 0.05)?	Yes
Data analyzed	
Sample size, group A	6
Sample size, group B	6

Number of values	6	6
Minimum	248754	274599
Maximum	275642	281321
Range	26888	6722
Mean	260530	278023
Std. Deviation	12425	2530
Std. Error of Mean	5072	1033

LOD and LOQ values determinations for both matrices
Briefly, to calculate the LOD and LOQ values for the PIC product, two stock solutions for PIC were prepared in concentrated DCM extracts of each soil matrix (VA and Silt). Dilution of these solutions with additional, corresponding soil extracts until the S/N ratios fell between 5-20. The S/N ratios were calculated using the m/z = 85 ion (base peak for PIC). The values obtained were the following: Virginia type A extract: LOD: **4.9 ng/mL**; LOQ: **16.5 ng/mL**; Silt sediment: LOD: **8.3 ng/mL**; LOQ: **27.8 ng/mL**.

For the VA soil. **Two values are obtained for LOD and LOQ as these were determined using injections of PIC at concentrations of 0.1 and 0.5 ppm.**

Concentration (ppm)	PIC (m/z 85)-Rep 1	PIC (m/z 85)-Rep 2	PIC (m/z 85)-Rep 3	Avg	Std
0.05	58986	134801	139165	136983	3085.814
0.1	327421	679042	590986	532483	182965.2
0.5	3331945	3978181	4149977	3820034.333	431337.3
1	7865140	8549322	8958528	8457663.333	552426.7
10	100291620	104347889	104155743	102931750.7	2288438

			S/N (m/z 85)					LOD (ppm)	LOQ (ppm)
0.05	3312.3								
0.1	532483		45.4	67.9	68.5	60.6		0.0049505	0.016502
0.5	3820034		385.1	371.3	323.1	359.8333		0.0041686	0.013895
1	8457663								
10	1.03E+08								

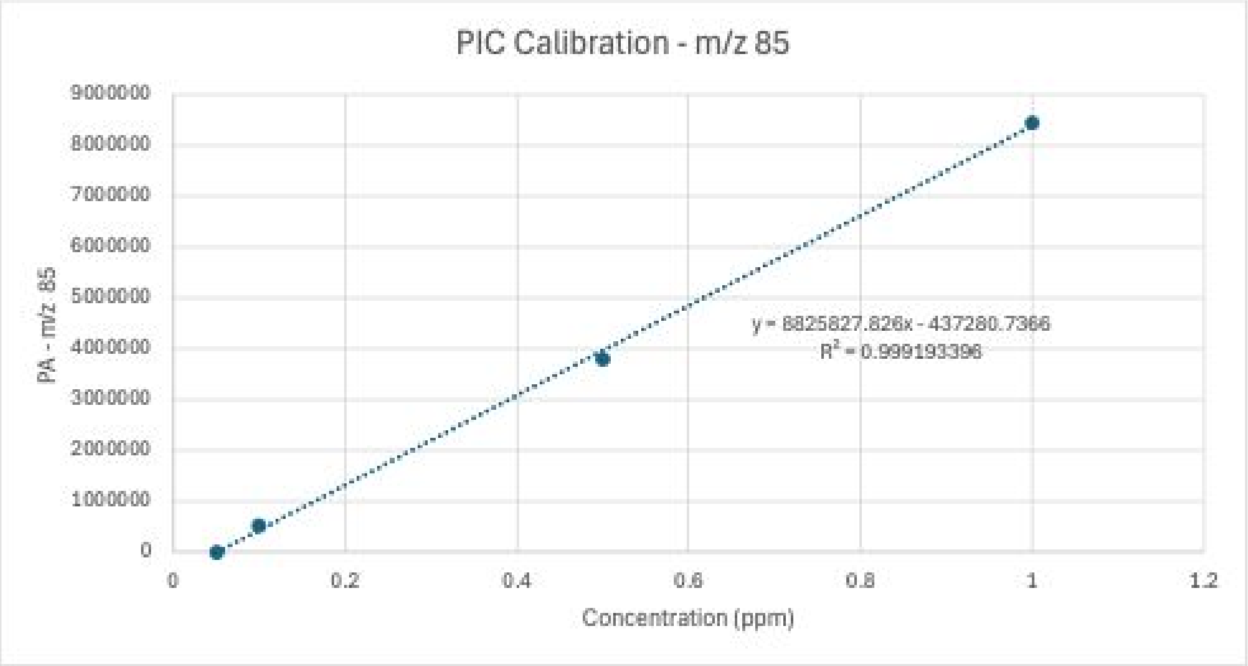

For the silt sediment. **Two values are obtained for LOD and LOQ as these were determined using injections of PIC at concentrations of 0.1 and 0.5 ppm.**

Concentration (ppm)	PIC (m/z 85)-Rep 1	PIC (m/z 85)-Rep 2	PIC (m/z 85)-Rep 3	Avg	Std
0.05	35665	36483	53489	41879	10062.87
0.1	194156	275559	277462	249059	47556.91
0.5	2389703	2114568	2483538	2329269.667	191765.1
1	4630305	5285062	5513774	5143047	458536.3
10	69908331	74919315	75701970	73509872	3143479

			S/N (m/z 85)					LOD (ppm)	LOQ (ppm)	
0.05	41879									
0.1	249059		28.4	25.3	54.3	36		0.0083333	0.027778	
0.5	2329270		185.3	185.2	212.9	194.4667		0.0077134	0.025711	
1	5143047									
10	73509872							LOD = 3 * Concentration (ppm)/(S/N)		
								LOQ = 10 * Concentration (ppm)/(S/N)		

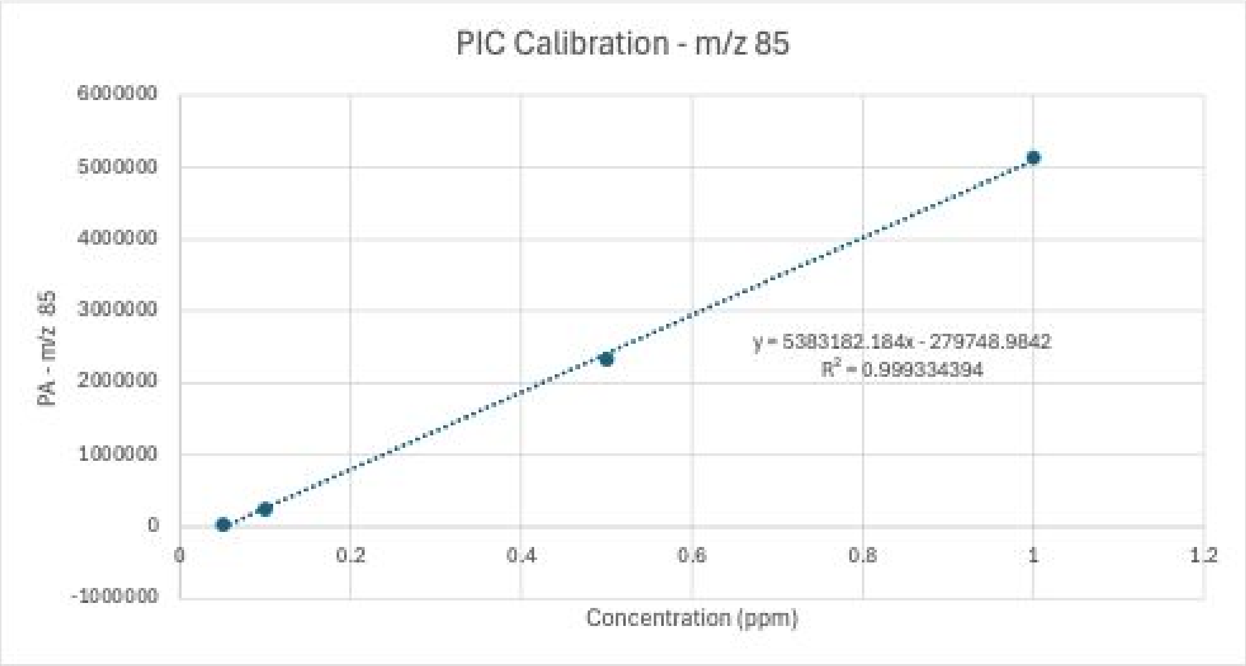

Comparative studies between silylation and carbamoylation of PA

A comparative study was carried out to assess the effectiveness of carbamoylation over two well-established and widely used silylation methods. The first one is trimethylsilylation using BSTFA while the second method is silylation of PA with a bulkier and therefore more stable group using phenyldimethylsilyl chloride (PDMSCl) and *N*-methylimidazole (NMI) (**Scheme S1**). The PA was spiked in a DCM extract of Sandy Loam soil at a concentration of 20 µg/mL. Each PA-spiked sample was then treated separately with excess CDI (5 mg), BSTFA (20 µL) and PDMSCl (20 µL) /NMI (35 µL) and reacted at 55 °C, 65 °C and RT for 3 h. The reaction mixtures were further diluted two-fold and analyzed directly by EI-GC-MS.

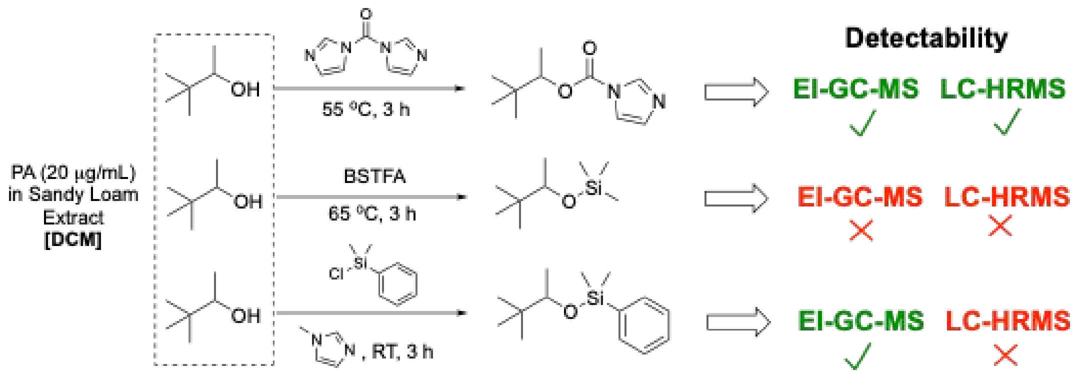

Scheme S1. Derivatization of PA separately using CDI, BSTFA and PDMSCl/NMIfor its analysis by EI-GC-MS and LC-HRMS. The PA was derivatized in a Sandy Loam DCM extract (~20 µg/mL) with each reagent as a comparative study among these three derivatization methods. The only product detectable by both techniques used in this work is the PIC arising from the carbamoylation reaction.

EI-GC-MS analysis were carried out in a new instrument, different from the one used in the previous analyses and this explains the difference in retention times obtained for the PIC product. All derivatizations were conducted in duplicates. As it can be seen in Figure S24, the PIC product can be easily detected in the Sandy Loam extract. The same cannot be stated for the PA-TMS product which is not detected by EI-GC-MS and interestingly there is no match for it in the NIST library (Figure S25). Lastly, the PDMS-PA derivative can be detected by EI-GC-MS (Figure S26) and its mass spectrum matches the one published for it [ref. 34].

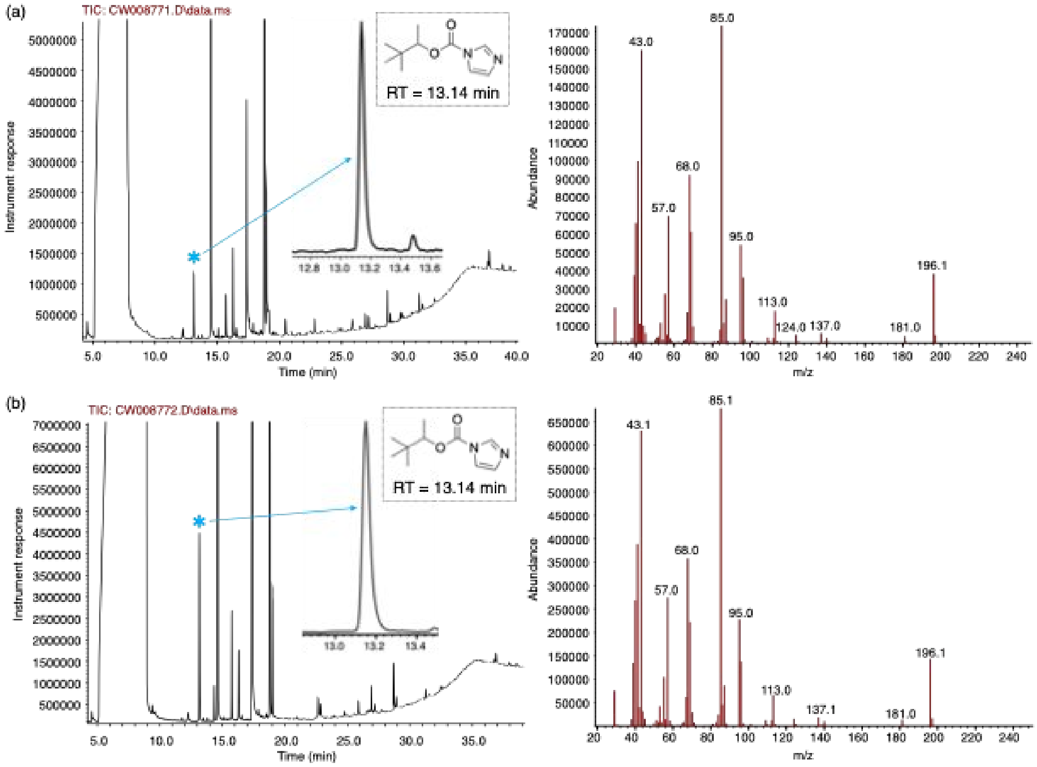

Figure S24. PIC formation from the CDI-derivatization of PA when spiked at 20 µg/mL in Sandy Loam extracts (a) and (b).

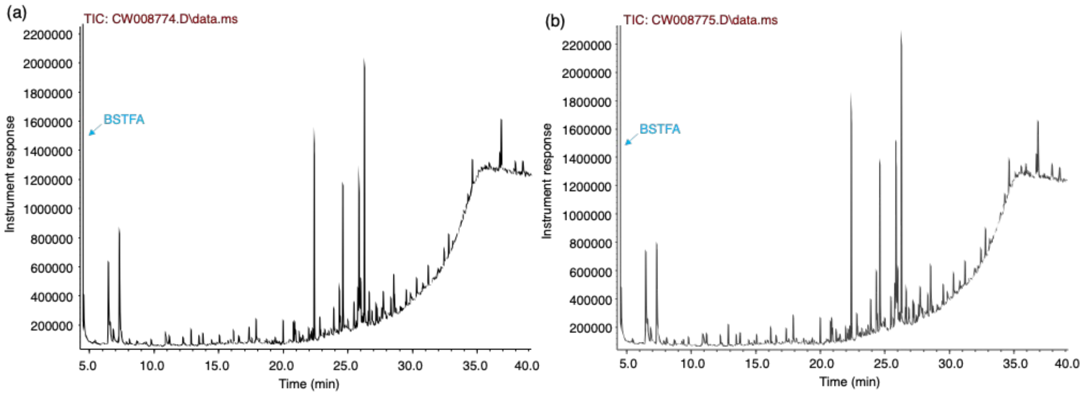

Figure S25. GC-MS analysis of the BSTFA-derivatized extracts spiked with PA at 20 µg/mL. No trace of the PA-TMS product was detected.

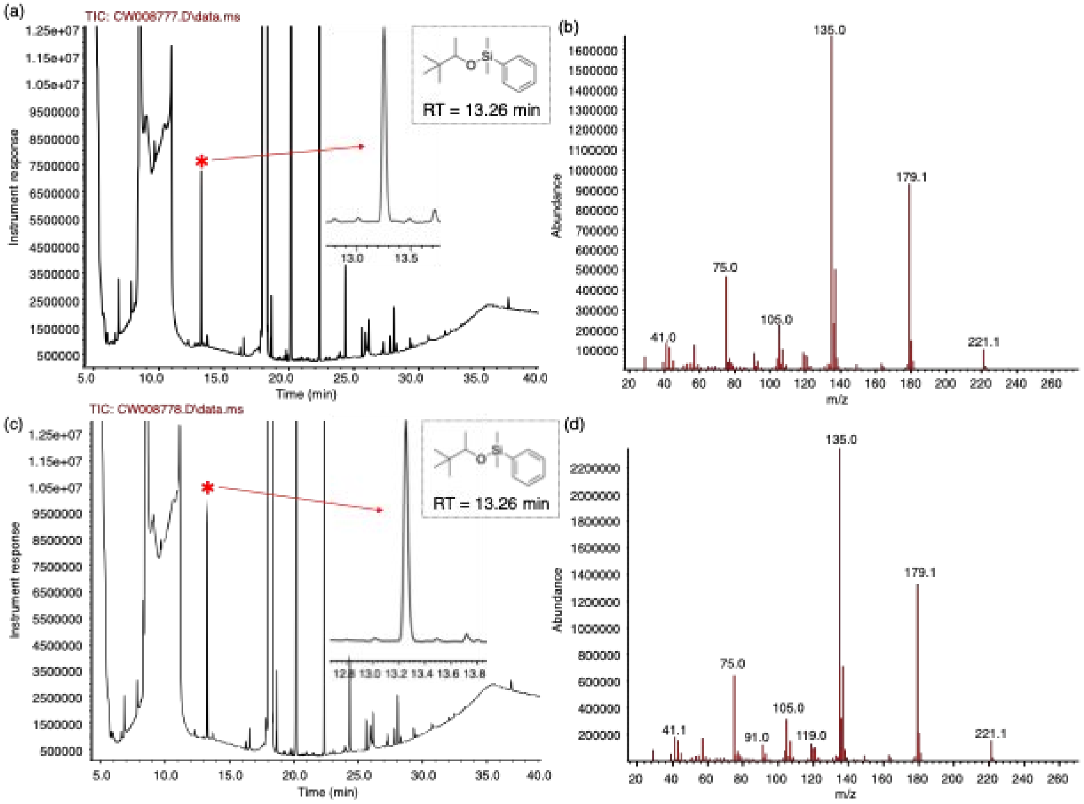

Figure S26. GC-MS analysis of the PDMSCl/NMI silylated extracts spiked with PA at 20 µg/mL. The PDMS-PA product was detected in both reactions.

LC-HRMS analysis of all three derivatization methods

The samples analyzed by EI-GC-MS above were also analyzed by LC-HRMS. As it can be appreciated in all three figures that follow, only the PIC product arising from the Carbamoylation can be detected by LC-HRMS (Figure S27), while no product is detected for PA-TMS (Figure S28) and the PA-PDMS (Figure S29). Even though it seems like both silylated analogs could be detected using the $[M+H]^+$ ion extraction mode, these results can not be considered as detection of the derivatives as their abundance is very low (i.e., E4). By comparison, the PIC product is detected quite easily, and it is prominent in the chromatogram (i.e., E8 intensity).

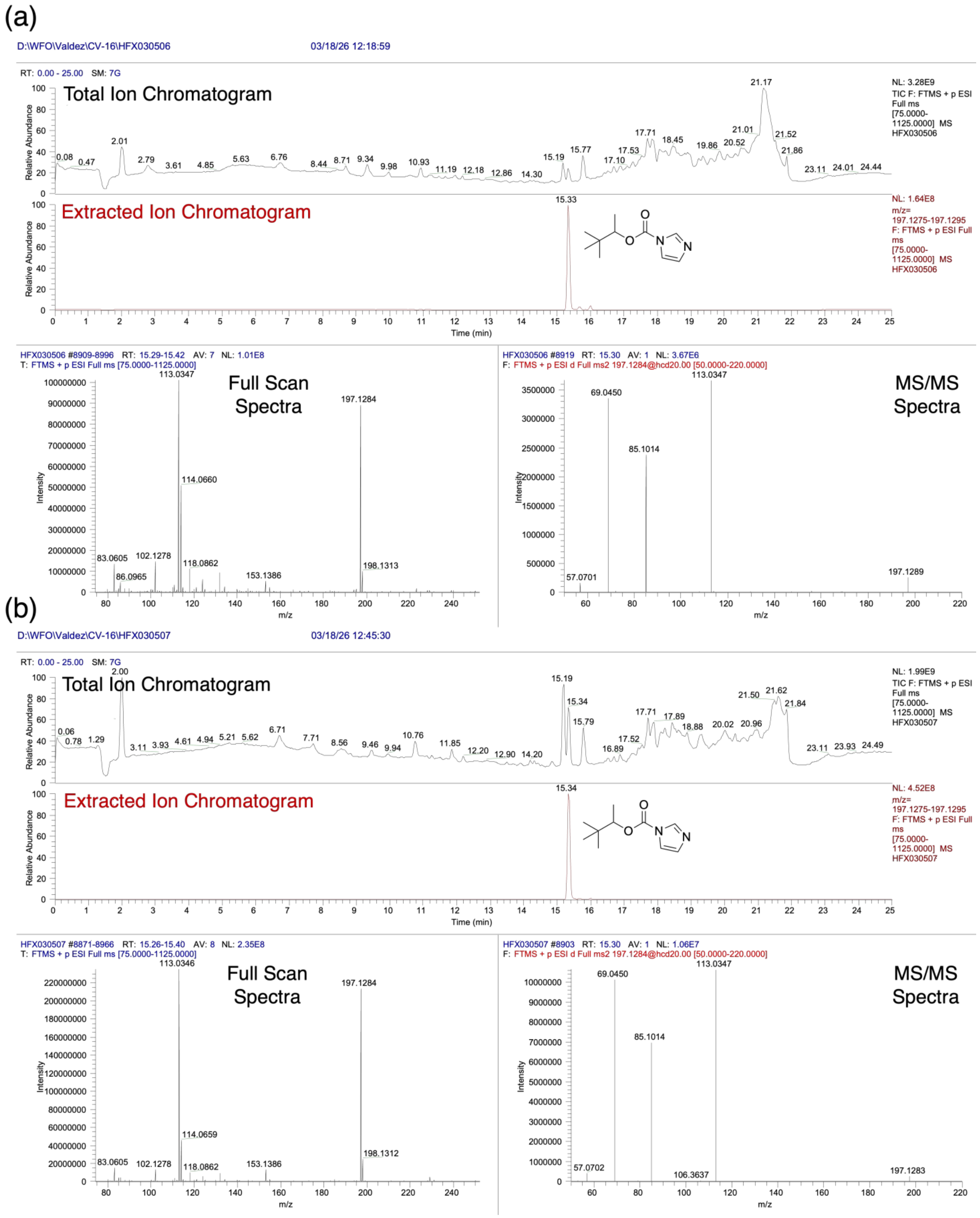

Figure S27. LC-HRMS analysis of the CDI-derivatized extracts (a) and (b) spiked with PA at 20 µg/mL.

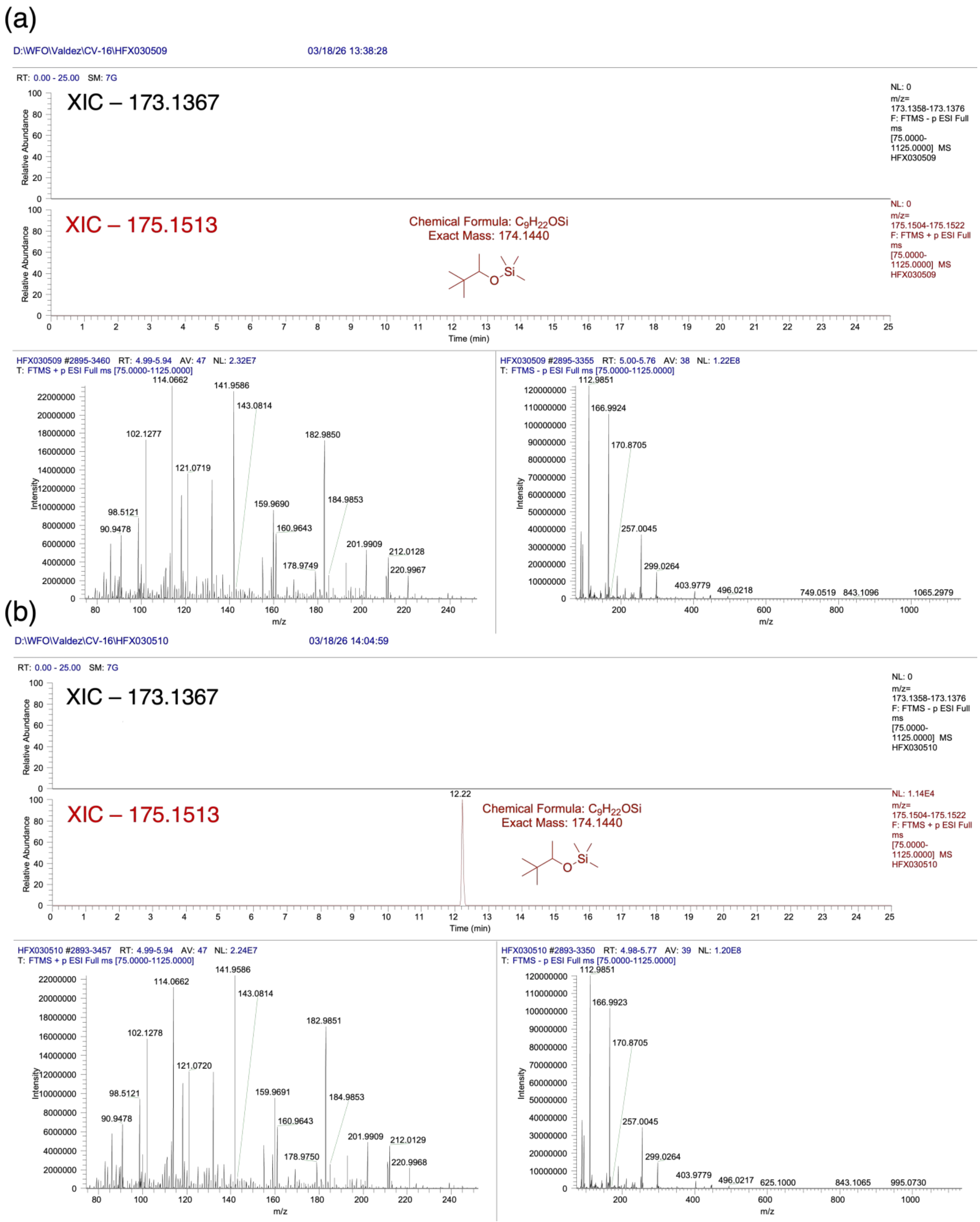

Figure S28. LC-HRMS analysis of the BSTFA-derivatized extracts (a) and (b) spiked with PA at 20 µg/mL. No detectable PA-TMS can be observed.

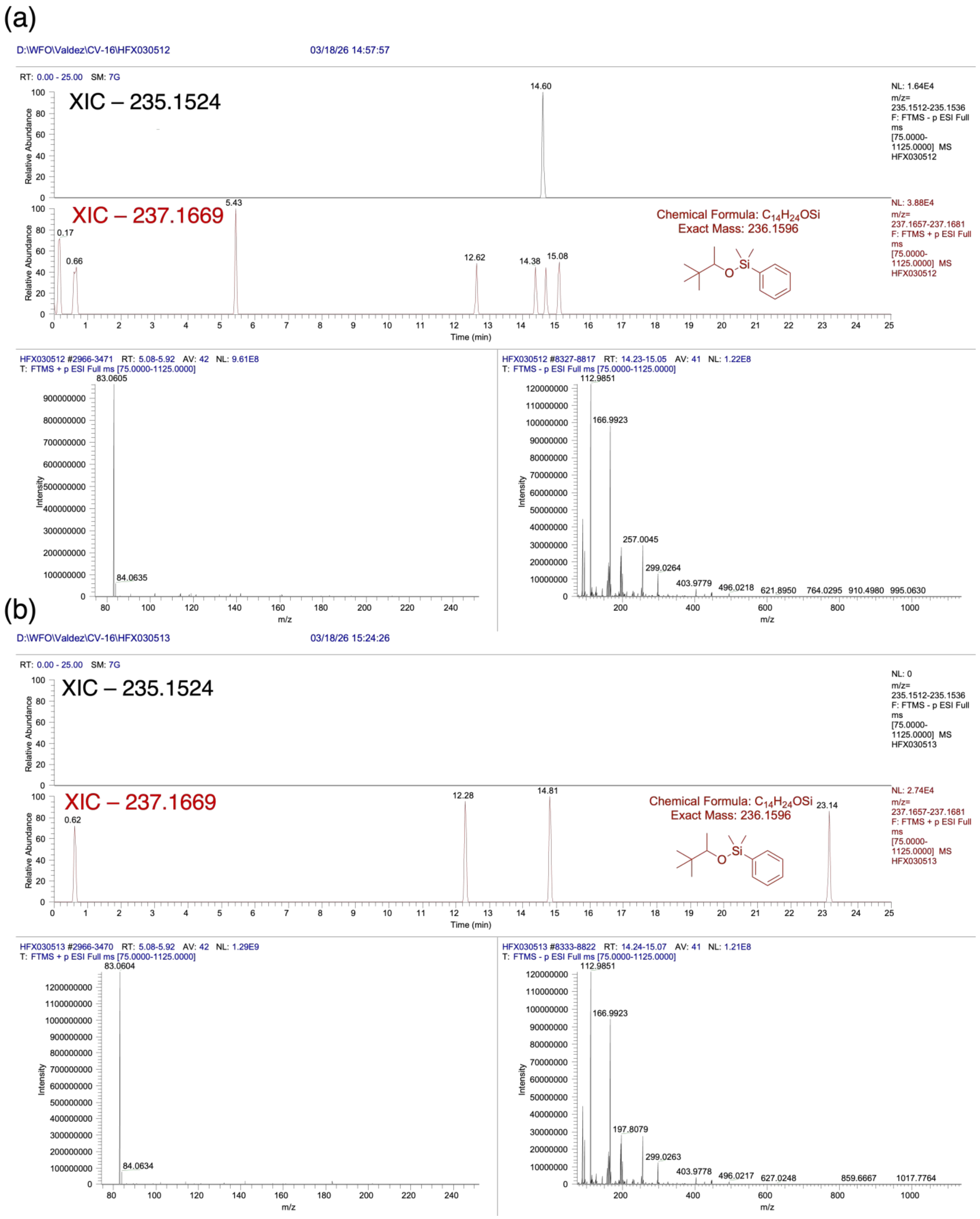

Figure S29. LC-HRMS analysis of the PDMS-derivatized extracts (a) and (b) spiked with PA at 20 μg/mL. No detectable PA-PDMS can be observed.
